# Upregulation of VEGFA through the adenosine A2A receptor is a crucial pathway for inhibiting pericyte apoptosis in chronic cerebral hypoperfusion

**DOI:** 10.1038/s41598-025-08407-2

**Published:** 2025-07-04

**Authors:** Deyue Li, Pan Gao, Wei Duan

**Affiliations:** 1 Department of Neurology, The Second Affiliated (Xinqiao) Hospital, The Army (Third Military) Medical University, Chongqing, 400037 China; 2 Department of Pharmacy, The Second Affiliated (Xinqiao) Hospital, The Army (Third Military) Medical University, Chongqing, China

**Keywords:** Chronic cerebral hypoperfusion, Pericyte, Adenosine A2A receptor, Vascular endothelial growth factor A, Apoptosis, Cell death in the nervous system, Cognitive neuroscience, Molecular neuroscience

## Abstract

Chronic cerebral hypoperfusion (CCH) is a key factor in vascular cognitive impairment. Pericyte loss and subsequent blood-brain barrier disruption play pivotal roles in the pathogenesis of CCH-induced white matter lesions (CCH-WMLs). Previous work suggested that the adenosine A2A receptor (A2AR) may protect pericytes in CCH-WMLs, but the mechanisms are not fully understood. In this study, we induced CCH in Sprague‒Dawley rats via bilateral carotid artery occlusion and treated them with the A2AR agonist CGS21680 or the A2AR antagonist SCH58261. Our findings revealed that CGS21680 significantly inhibited the expression of the proapoptotic proteins BAX and Caspase 3, while SCH58261 obviously promoted it. The expression of the antiapoptotic protein Bcl-2 was markedly increased by CGS21680 in OGD-exposed pericytes. Additionally, the expression of the transcription factors Rap-1, ERK, and phosphorylated ERK also increased dramatically in OGD-exposed pericytes following CGS21680 administration. VEGFA and VEGFR2 expression was upregulated by CGS21680 and downregulated by SCH58261 in pericytes after OGD. Furthermore, VEGFA knockdown via a shRNA-expressing adenovirus counteracted the protective effect of A2AR against pericyte apoptosis following OGD. Notably, the expression of BAX and Caspase3 was significantly upregulated, and the expression of BCL-2 was markedly downregulated in OGD-exposed pericytes after Rap-1 knockdown via a shRNA-expressing adenovirus. Rap-1 suppression obviously reduced the levels of phosphorylated ERK, VEGFA and VEGFR2 in pericytes, suggesting a role for the Rap1-ERK pathway in the A2AR-induced upregulation of VEGFA expression. Overall, A2AR activation inhibits pericyte apoptosis and may exert neuroprotective effects against CCH by increasing VEGFA expression through the Rap1-ERK signaling pathway.

**Supplementary Information:**

The online version contains supplementary material available at 10.1038/s41598-025-08407-2.

## Introduction

Chronic cerebral hypoperfusion (CCH) is regarded as a significant contributor to vascular cognitive impairment (VCI)^1–3^. While a consensus on the pathology of VCI is currently lacking, diffuse white matter lesions (WMLs) are recognized as a significant pathological feature of vascular dementia^4^. The integrity of the white matter is critical for efficient neuronal communication and the maintenance of cognitive function. Therefore, the disruption of white matter tracts can impair communication between different brain regions and contribute to cognitive impairment. Although several mechanisms have been reported contributing to VCI, CCH-induced WMLs (CCH-WMLs) have been proposed as the central underlying cause^5,6^. However, the mechanism of CCH-WML development remains to be elucidated, and effective treatments are currently lacking.

Recent studies have consistently revealed significant disruption of the blood-brain barrier (BBB) during the initial stages of CCH^7,8^. BBB disruption is believed to contribute to the progression of white matter injuries, further aggravating cognitive impairment^9^. As integral constituents of the neurovascular unit, pericytes play a pivotal role in maintaining the integrity of the BBB^10^. They are acknowledged for their diverse functions^11^, such as contributing to the structural integrity of the BBB, undergoing contraction, releasing cytokines that enhance communication with endothelial cells, and promoting cell differentiation through their stem-like properties. Pericytes have been noted to significantly decrease in number and show functional impairment in central nervous system diseases, particularly in conditions characterized by CCH^12,13^. Additionally, a marked decline in hippocampal capillary pericytes has been noted in VCI^14^, coupled with contractile dysfunction that further narrows capillaries, exacerbating the damage caused by CCH^15^. However, the mechanisms contributing to pericyte loss in CCH have not yet been fully elucidated. Further research is imperative to identify these processes, which could help researchers discover new therapeutic targets for treating VCI.

In recent years, the association between the adenosine A2A receptor (A2AR) and the pathophysiology of central nervous system (CNS) disorders has become clearer, and A2AR has been identified as a potential therapeutic target for a range of CNS conditions, such as ischemic stroke^16^, Alzheimer’s disease, and Parkinson’s disease^17^. Our preliminary findings suggest that the absence of A2AR exacerbates white matter damage caused by CCH^18^. Further research has indicated that A2AR ameliorates this damage by promoting the M2 polarization of microglia, thereby reducing neuroinflammatory responses^19^. However, the exact mechanism through which A2AR attenuates white matter injury in conditions involving CCH remains fully elucidated. Numerous studies have confirmed that A2AR plays a key role in protecting against BBB dysfunction^20–22^. More importantly, more studies have revealed that A2AR reduces BBB damage induced by CCH by relieving pericyte dysfunction^20,23^. Thus, pericytes are a potential target of A2AR in treating CCH-induced white matter injuries. However, the underlying mechanism by which A2AR regulates pericyte function to attenuate hypoperfusion-induced white matter damage remains to be elucidated. In this study, we utilized a rat model of CCH induced by bilateral carotid artery occlusion and pericytes subjected to oxygen-glucose deprivation (OGD) to confirm the finding about pericyte loss in CCH and explore the regulatory role of A2AR in the pathological process. Additionally, in vitro studies were performed to delineate the signaling pathways through which A2AR suppresses pericyte apoptosis in CCH conditions.

## Materials and methods

### Animals

All animal experiments were performed following the recommendations of the Guide for the Care and Use of Laboratory Animals of the National Institutes of Health and reported following the ARRIVE (Animal Research: Reporting In Vivo Experiments) guidelines. Male Sprague‒Dawley (SD) rats, weighing 200–300 g and aged eight weeks, were procured from ENSIWEIER Biotechnology Co., Ltd (Chongqing). The animals were housed under standardized conditions with a temperature range 20–25℃ and a 12-hour light/dark cycle. They had unrestricted access to water and food.

## Bilateral carotid artery occlusion surgery

The rat model of permanent bilateral occlusion of the common carotid arteries (2VO) was established using established methods^24^. Briefly, animals were anesthetized with pentobarbital (50 mg/kg), and a midline incision was made to expose and dissect the common carotid arteries away from the vagus nerve. Subsequently, the bilateral common carotid arteries were successively ligated with 4 − 0 silk sutures. Intraoperatively, rectal temperature was maintained at 37 ± 0.5℃. After surgery, electric blankets are used to maintain body temperature until the rats wake up. After surgery, adequate food and water are provided, and an appropriate amount of glucose is added to the water to replenish energy. At the same time, rats’ mental state, activity ability, and appetite should always be observed. Brain tissues were collected at 3,7,14, and 28 days post-surgery for experimental analysis. A 30% mortality rate was observed within 24 h post-surgery, possibly due to anesthesia overdose or surgical trauma. The Institutional Animal Care and Use Committee of the Army Medical University (SYXK-PLA-2007035) provided ethical approval for all animal care and experimental protocols, which were conducted in accordance with the 3Rs principles (Replacement, Reduction, and Refinement). Researchers conducting evaluations were blinded to the treatment groups.

## Adenosine A2A receptor agonist and antagonist treatment

The adenosine A2A receptor (A2AR) agonist CGS21680 (HY-13201, 50 mg, MCE) and antagonist SCH58261 (HY-19533, 50 mg, MCE) were dissolved in a vehicle of 10% dimethyl sulfoxide (DMSO) and 90% corn oil. Rats were randomly assigned to the 2VO-Saline group, 2VO-SCH group and 2VO-CGS group following the 2VO operation. A2AR agonist/antagonist treatment was performed on the second day after surgery. The 2VO-SCH group and 2VO-CGS group received daily intraperitoneal injections of SCH58261 (0.1 mg/kg)^25,26^ and CGS21680 (0.1 mg/kg)^27,28^, respectively. The 2VO-Saline group was administered an equal volume of saline as a control. All injections were performed in the afternoon between 3:00 PM and 6:00 PM.

## Pericyte cell culture and oxygen-glucose deprivation (OGD) establishment

Primary rat brain pericytes were procured from Meisen Cell (CTCC-N003-MIC, Zhejiang, China) and cultured in modified eagle medium (MEM) supplemented with 10% FBS and 1% penicillin/streptomycin. Cells were maintained in a humidified incubator at 37℃ with 95% air and 5% CO_2_. Pericytes (1 × 10^6 per well) were divided into three groups: OGD group, OGD + CGS group and OGD + SCH group. The OGD + CGS group was treated with A2AR agonist CGS21680 (1 µM)^23,29^, and the OGD + SCH group was added with antagonist SCH58261 (1 µM)^23^. The OGD group was administered an equal volume of MEM as a control. After half an hour, the treated cells were transferred to low-glucose MEM under a hypoxic atmosphere (3% O_2_, 5% CO_2_ and 92% N_2_) for 18 h, as described previously^19^.

## Adenoviral transfection with Rap1 or VEGFA shRNA

A biotechnology company constructed a small hairpin RNA (shRNA) adenovirus for VEGFA. The interference efficiency was detected using western blot and polymerase chain reaction (PCR). Pericytes were seeded in 6-well plates and allowed to reach approximately 40% confluence. A shRNA transfection mixture containing VEGFA-shRNA and lip2000 was applied, and the pericytes were incubated for 24 to 72 h. Following successful transfection, pericytes were categorized into the OGD + CGS group, OGD + CGS + V group, and OGD + CGS + VEGFA-shRNA group. A2AR agonist CGS21680 (1 µM) was added to each group half an hour after successful transfection. Subsequently, the above three groups of pericytes were subjected to hypoxic conditions in low-glucose MEM for 18 h.

Consistent with the above procedure, pericytes were also successfully transfected by a shRNA adenovirus for Rap-1 and an empty vector. Half an hour after successfully transfection, A2AR agonist CGS21680 (1 µM) was also administered to each group. Pericytes were categorized into the OGD + CGS group, the OGD + CGS + V group, and the OGD + CGS + Rap-1-shRNA group. Lastly, all groups were subjected to hypoxic conditions in low-glucose MEM for 18 h.

### Immunofluorescence staining and TUNEL assay

Immunofluorescence staining was used in both in vitro and in vivo experiments. Platelet-derived growth factor receptor β (PDGFRβ) was used to label pericytes. Terminal deoxynucleotidyl transferase-mediated dUTP nick-end labeling (TUNEL) assay was used to detect apoptotic cells.

Firstly, model rats in each group were euthanized by intraperitoneal injection of lethal dose of pentobarbital(150 mg/kg) and cardiac perfusion was paraformaldehyde at 3, 7, 14, and 28 days post-surgery. Brains were extracted and fixed in 4% paraformaldehyde at 4℃ for 24 h and dehydrated in 30% sucrose at 4℃ for 72 h. Coronal brain Sect. (20 μm thick) were cut, mounted on APS-coated slides, and stored at −80℃. The serial coronal sections were preincubated in 5% goat serum and incubated overnight at 4℃ in the primary antibody solution for PDGFRβ (1:100, 3169 T, CST, USA) followed by three-time washes in the PBS. After washing, fluorescein isothiocyanate FITC-conjugated goat anti-rabbit secondary antibodies (1:50, ZF0311; ZF0316, ZhongshanJinQiao Biotechnology Co. Ltd, Beijing, China) were applied, and samples were incubated at room temperature for 1 h. TUNEL assay was performed following the instructions provided. TRITC-dUTP labeling Mix was used to mark apoptotic cells. All sections were counterstained with 4’,6-diamidino-2-phenylindole (DAPI, 40 mg/ml) for 5 min and visualized using confocal microscopy. For confocal microscopy detection, 200×non-overlapping high-power fields (0.5 × 0.5 mm, total area of 0.25 mm^2^) in the center of the corpus callosum were selected using a square grid inserted into the eyepiece. The ratio of PDGFRβ positive cells to TUNEL-positive cells was speculated and compared between the groups. Three fields within the regions of interest in three sections per animal were examined. For each section, the ratio in three selected fields of the corpus callosum was averaged.

Secondly, the treated pericytes were detected by TUNEL assay with TRITC-dUTP labeling Mix to detect apoptotic cells. After staining, a Leica fluorescence microscope was used to examine each cell section, and 200×non-overlapping high power fields (0.5 × 0.5 mm, total area of 0.25 mm^2^) in the center of the eyepiece were selected. The number of TUNEL-positive cells in these regions was counted. Three fields within the regions of interest were examined in three slices from each group. For each slice, the ratios of positive cells to 0.25 mm^2^ in three selected fields were averaged.

## Flow cytometry

Apoptosis was also assessed using the Annexin V-FITC Apoptosis Detection Kit (C1062S, Beyotime, Shanghai, China). Pericytes in each group were seeded in 6-well plates and exposed to OGD conditions for 18 h after CGS21680 or SCH58261 treatment. Cells were harvested, resuspended in binding buffer, and stained with Annexin V-FITC and propidium iodide. Samples were analyzed using a flow cytometry, and data were processed with Flowing Software.

## Quantitative real-time polymerase chain reaction detection (RT-PCR)

Total RNA was isolated from pericytes in OGD + CGS group, OGD + CGS + V group and OGD + CGS + Rap-1-shRNA group using Trizol reagent. Total RNA was reversely transcribed using MMLV Reverse Transcriptase (Thermo Fisher Scientific, USA). Complementary DNA was amplified by PCR using a SYBR Green kit (TaKaRa BioInc, Dalian, China) and an ABI 7500 qPCR system (Applied Biosystems). Each PCR cycle consisted of 5 min of denaturation at 95℃, 30 s of denaturation at 95℃, 40 s of annealing at 65℃ and 40 s of extension at 72℃, and 42 PCR cycles were performed. β-actin was used as the internal control. The 2^−△△ct^ method was used for the quantification. The sequences of the primers used for VEGFA, VEGFR2 and β-actin were exploited: forward primer: 5’-CTCACACACACACCAACCAGG-3’, reverse primer:5’-GAAGAAGCAGCCCATGACAG-3’, VEGFR2: forward primer:5’-CGGACAGTGGTATGGTTCTTGC-3, reverse primer:5’-GTGGTGTCTGTGTCATCGGAGTG-3; β-actin: forward primer: 5’-AGCGAGCATCCCCCAAAGTT-3, reverse primer: 5’-GGGCAC GAAGGCTCATCATT-3. For each sample, at least three independent PCR experiments were performed.

### Western blot analysis

Western blot analysis was also used both in vitro and in vivo experiments. Total protein was extracted from cells using a whole protein extraction kit (Key GEN, China). Total protein concentrations were determined on a UV spectrophotometer using a modified Bradford assay (Beckman Coulter, Fullerton, CA; Ran et al.,2015). Equal amounts of protein from each sample were separated via electrophoresis on 8% polyacrylamide gels and then transferred to polyvinylidene fluoride (PVDF) membranes. The membranes were blocked by 5% skimmed milk and incubated overnight at 4◦C with the following primary antibodies: rabbit anti-VEGFA antibody (1:500, AF5131, Affinity Bioscience, Changzhou, China), rabbit anti-VEGFR2 antibody (1:500, AF6281, Affinity Bioscience, Changzhou, China), rabbit anti-BAX antibody (1:500, AF0120, Affinity Bioscience, Changzhou, China), rabbit anti-BCL-2 antibody (1:500, AF6139, Affinity Bioscience, Changzhou, China), mouse anti-Caspase 3 antibody (1:500, 66470-2-Ig, Proteintech, Wuhan, China), rabbit anti-Rap-1 antibody (1:500, AF6147, Affinity Bioscience, Changzhou, China), rabbit anti-PKA antibody (1:1000, AF7746, Affinity Bioscience, Changzhou, China), rabbit anti-P-PKA antibody (1:500, AF7246, Affinity Bioscience, Changzhou, China), rabbit anti-ERK antibody (1:1000, 4695 S, CST, USA), or rabbit anti-P-ERK antibody (1:1000, 4370 S, CST, USA). After being washed with TBST three times, the membranes were incubated with goat anti-rabbit secondary antibody (1:1000, ZB2301, ZSGB-Bio, Beijing, China) or goat anti-mouse secondary antibody (1:100, ZB2305, ZSGB-Bio, Beijing, China). The amount of β-actin (1:2000, SC-47778, Santa Cruz Biotechnology, Santa Cruz, CA) or Tubulin (1:1000, AF7010, Affinity Bioscience, Changzhou, China) was used as the internal control. The PVDF membranes were developed and visualized, and the optical density (OD) of each specific protein band was measured using an image analysis software (QuantityOne 4.4.0.36; Bio-Rad, Hercules, CA) and normalized to the OD of the β-actin or Tubulin.

### Enzyme-linked immunosorbent assay (ELISA)

VEGFA expression in model rats and OGD-cultured pericytes was examined by ELISA after an agonist or antagonist was administrated. Rat Quantikine ELISA kits for VEGFA (JL21370, JONLNBIO, Shanghai, China) were used in the study. Working reagents were prepared, and 50 µl of standard controls and cell supernatants from each group were added to the wells of a plate according to manufacturer instructions. The antibody was added and incubated for two hours at room temperature, followed by incubation with the substrate solution for 30 min. The absorbance was read using a FLvostar Omega microplate reader at 450 and 540 nm.

### Statistical analysis

Data are presented as mean ± SEM from three independent experiments, each performed in triplicate. Normality and variance homogeneity were assessed. One-way ANOVA with Bonferroni or Tamhane T2 post-hoc analysis was used for multiple comparisons, while two-way ANOVA with Turkey’s multiple comparisons test was applied for two-factor analysis. When the experimental data does not follow a normal distribution, the Mann-Whitney U test was used for two independent samples, and the Kruskal-Wallis H test was used for multiple independent samples. All of the data used for the analysis were viewed in a blinded manner. The data were plotted and analyzed with GraphPad Prism 6. *P* < 0.05 was considered a statistical difference, and *P* < 0.01 was considered statistically significant.

## Results

### Adenosine A2AR agonists inhibit pericyte apoptosis in a rat model of CCH

**Fig. 1:**
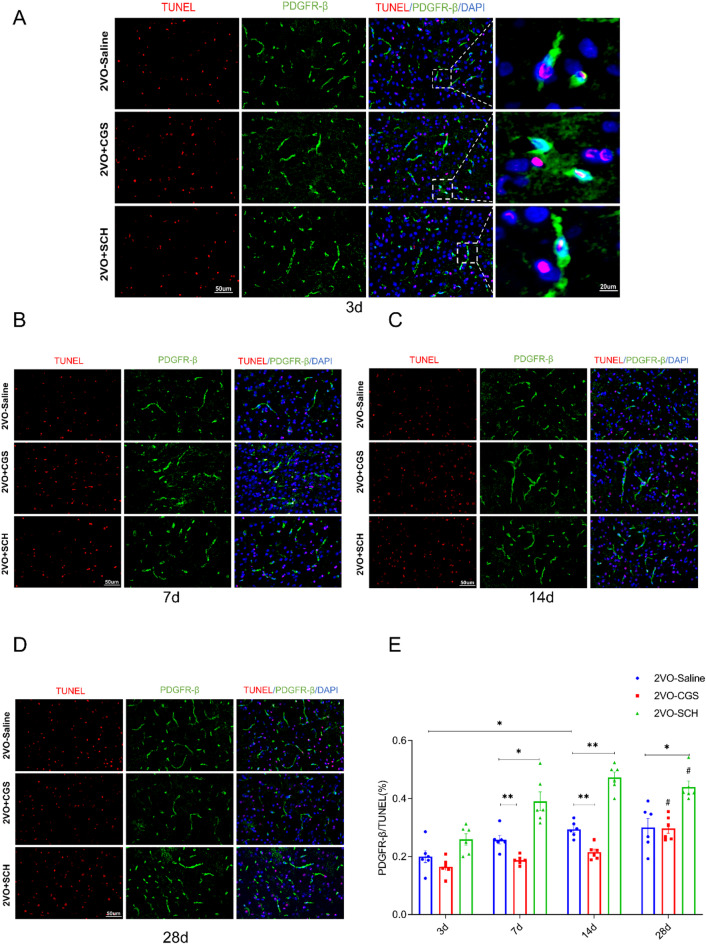
Adenosine A2A receptor inhibition of brain pericyte apoptosis in a rat model of chronic cerebral hypoperfusion. (**A**-**D**) Immunofluorescence staining illustrates pericytes (green) and apoptotic cells (red) at 3, 7, 14, and 28 days post-bilateral common carotid artery occlusion (2VO). (**E**) Histogram represents the ratio of PDGFRβ positiveto TUNEL-positive cell counts at different time point. A significant increase in this ratio at 14 days compared to 3 days post-2VO is observed in the 2VO-Saline group. In contrast, the 2VO-CGS group showed a significant reduction in the ratio at 7 and 14 days post-2VO (*P* < 0.01), indicating an inhibitory effect of CGS21680 on pericyte apoptosis. Conversely, the 2VO-SCH group exhibited a significant increase in the ratio at the same time points compared to the 2VO-Saline group (*P* < 0.05), suggesting that SCH58261 promotes pericyte apoptosis. Each group includes *n* = 5 samples. * Indicates *P* < 0.05, ** Indicates *P* < 0.01, #: *P* < 0.05, compared with 3d. Scale bars: 50 μm for the left four images, 20 μm for the right two images.

We examined the impact of adenosine A2AR agonists on pericyte apoptosis in an SD rat model of CCH induced by two-vessel occlusion (2VO). Immunofluorescence staining of a pericyte marker and staining of apoptotic cells were performed at 3, 7, 14, and 28 days after 2VO. PDGFRβ was utilized as a pericyte marker, and TUNEL staining was performed to identify apoptotic cells. Figure 1(A-D) shows representative images of double immunofluorescence staining in each group at the indicated time points after 2VO. As depicted in Fig. 1E, statistical analysis revealed significant differences in the PDGFRβ/TUNEL-positive cell count ratio among the treatment groups (*P* < 0.05). Specifically, the ratio at 14 days after CCH was significantly greater than that at 3 days after CCH in the 2VO-saline group, indicating increased pericyte apoptosis after CCH. Notably, at 7 and 14 days after 2VO, the PDGFRβ/TUNEL-positive cell count ratio was significantly lower in the 2VO-CGS group than in the 2VO-saline group (*P* < 0.01), suggesting that CGS21680 inhibits pericyte apoptosis. Conversely, the ratio was significantly greater in the 2VO-SCH group than in the 2VO-saline group (*P* < 0.05), indicating that SCH58261 promotes pericyte apoptosis in the context of CCH.

### Effect of A2AR on the protein expression of vascular endothelial growth factor A (VEGFA) and its receptor in a rat model of CCH

**Fig. 2:**
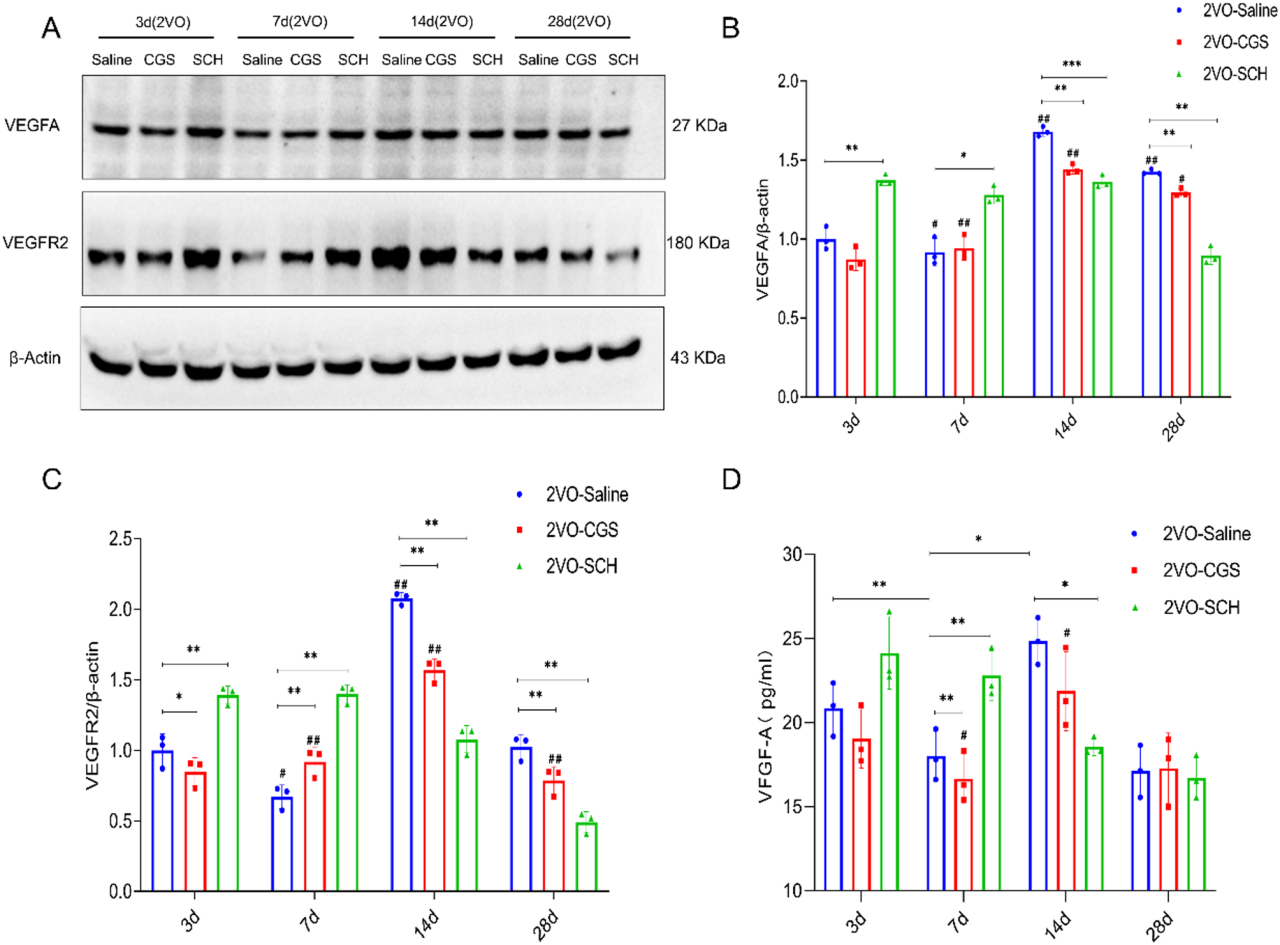
VEGFA and VEGFR2 expression dynamics in response to A2AR modulation in chronic cerebral hypoperfusion. (**A**-**C**) Western blot analysis was conducted to assess VEGFA and VEGFR2 protein expression at 3, 7, 14, and 28 days post-chronic cerebral hypoperfusion. (**A**) Protein bands for VEGFA and VEGFR2 are displayed. (**B**-**C**) Corresponding bar graphs represent the optical density values for VEGFA and VEGFR2 protein bands in each experimental group. (**D**) Bar graphs present the ELISA data analysis for VEGFA protein levels across the same time points. Each group comprised *n* = 5 samples. * Indicates *P* < 0.05, ** Indicates *P* < 0.01. #: *P* < 0.05, compared with 3 d, ##: *P* < 0.01, compared with 3d.

Using western blot analysis, we characterized the expression patterns of VEGFA and its receptor VEGFR2 in a rat model of CCH (Fig. 2A). Figure 2B shows the change in the OD ratio of VEGFA to β-actin over time, which peaked at 14 days after 2VO in the 2VO-saline group and significantly decreased by day 28 (*P* < 0.01). In rats treated with CGS21680, an A2AR agonist, the OD ratio of VEGFA to β-actin stabilized at 14 and 28 days and was significantly different from that in the 2VO-saline group (*P* < 0.01). Conversely, SCH58261, an A2AR antagonist, induced an early increase in the OD ratio of VEGFA to β-actin; specifically, the ratio increased at 3 and 7 days (*P* < 0.05), peaked at 14 days, and decreased by day 28 (*P* < 0.01). Notably, the OD ratio of VEGFA to β-actin was significantly reduced in the 2VO-CGS and 2VO-SCH groups compared with the 2VO-saline group at 14 and 28 days (*P* < 0.01).

To corroborate the western blot findings, ELISA was performed to quantify VEGFA protein levels following treatment with CGS21680 or SCH58261 (Fig. 2D). ELISA confirmed that VEGFA expression peaked in the 2VO-saline group at 14 days after 2VO and subsequently decreased by day 28 (*P* < 0.01). VEGFA expression in the 2VO-CGS group was significantly greater at 14 days than at 3 days after 2VO (*P* < 0.01). In contrast, VEGFA expression in the 2VO-SCH group was considerably lower at 14 and 28 days than at 3 days after 2VO (*P* < 0.01).

The VEGFR2 expression pattern mirrored the VEGFA expression pattern, with VEGFR2 expression peaking at 14 days (Fig. 2C, *P* < 0.01) and decreasing by day 28 in both the 2VO-saline and 2VO-CGS groups (*P* < 0.01). In the 2VO-SCH group, the OD ratio of VEGFR2 to β-actin sharply decreased at 14 and 28 days (*P* < 0.01). The OD ratio of VEGFR2 to β-actin in the 2VO-CGS group was significantly greater than in the 2VO-saline group at 7 days after 2VO (*P* < 0.01). Compared with that in the 2VO-saline group, the OD ratio of VEGFR2 to β-actin in the 2VO-SCH group significantly increased at 3 and 7 days after 2VO and decreased at 14 and 28 days after 2VO (*P* < 0.01).

### Initial experiment: temporal analysis of pericyte apoptosis after OGD

**Fig. 3:**
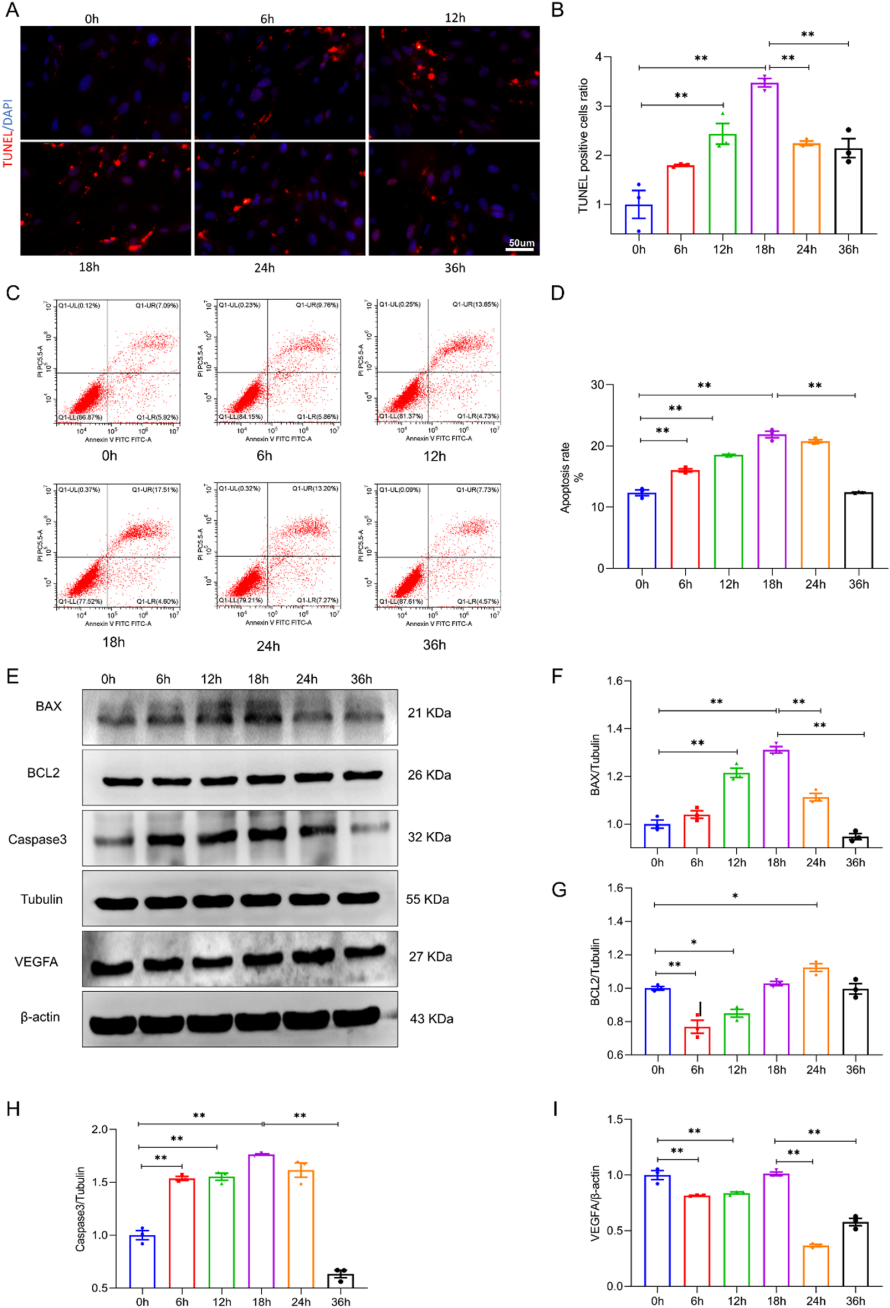
Dynamic apoptotic response of pericytes to oxygen-glucose deprivation. (**A**) Fluorescence microscopy images illustrate TUNEL-positive apoptotic cells (red) and DAPI-stained nuclei (blue) in pericytes at 0, 6, 12, 18, 24, and 36 h post-OGD induction. (**B**) Quantitative analysis of TUNEL-positive cells expressed as a ratio to total cells at various time points post-OGD induction. (**C**-**D**) Flow cytometry data depict the apoptotic rate of pericytes over time. (**E**-**I**) Western blot analysis of apoptosis-associated proteins (BAX, BCL-2, Caspase3) and VEGFA at various time points post-OGD condition. Quantitative assessment of TUNEL staining, flow cytometry, and western blot results collectively indicated that pericyte apoptosis was most pronounced at 18 h following OGD induction. Scale bars: 50 μm. * Indicates *P* < 0.05, ** Indicates *P* < 0.01.

We quantified pericyte apoptosis at multiple time points (0, 6, 12, 18, 24 and 36 h) following exposure to OGD. Through TUNEL staining and flow cytometry (Fig. 3A and C), we observed a progressive increase in TUNEL-positive cells, with a significant peak at 18 h after OGD exposure (Fig. 3B and D, *P* < 0.05), followed by a decrease at 24 and 36 h. Western blot analysis revealed the same biphasic pattern in the expression levels of the apoptosis-related proteins BAX, BCL-2, and Caspase3, with the changes in expression peaking at 18 h after OGD (Fig. 3E, F, G and H; *P* < 0.05). Additionally, VEGFA protein expression initially decreased but then increased at 18 h after OGD, indicating a dynamic regulatory response to OGD (Fig. 3I, *P* < 0.05). Collectively, these results identify 18 h after OGD as a critical peak of pericyte apoptosis.

### Effects of modulating A2AR expression and VEGFA-VEGFR2 expression changes in apoptotic pericytes after OGD exposure

**Fig. 4:**
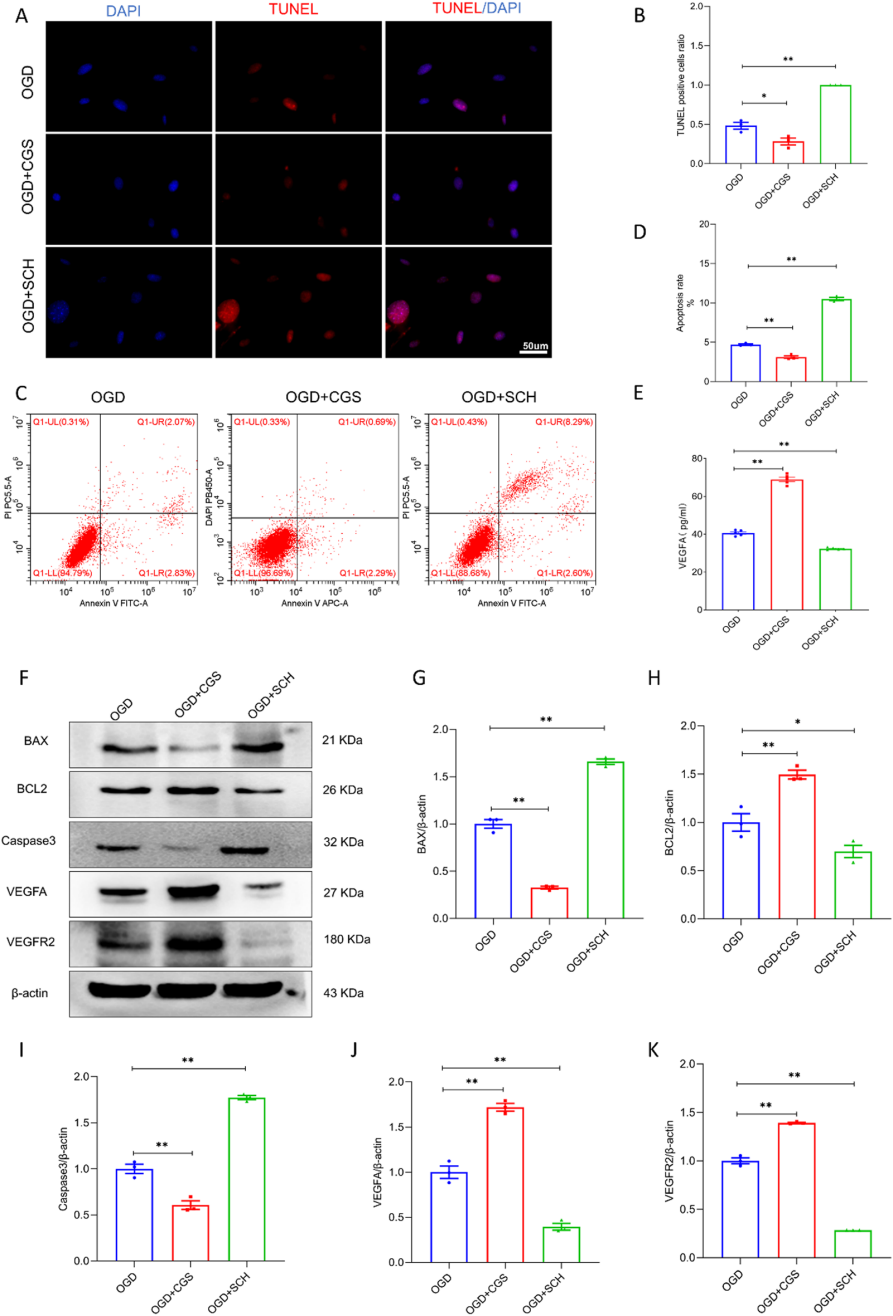
Adenosine A2AR agonist mitigates pericyte apoptosis at 18 h following oxygen-glucose deprivation. (**A**) Fluorescence microscopy captures TUNEL-positive apoptotic pericytes (red) and DAPI-stained nuclei (blue) in OGD group, OGD + CGS group and OGD + SCH group at the 18-hour time point post-OGD. (**B**) The bar graph displays the ratio of TUNEL-positive cells in each group at 18 h post-OGD. (**C**-**D**) The pericyte apoptotic rates detected by flow cytometry analysis in each group at the 18 h under OGD conditions. (**E**-**K**) Western blot analysis of apoptosis-associated proteins (BAX, BCL-2, Caspase3) and VEGFA-VEGFR2 signaling molecules in each group at 18 h post-OGD. The data collectively demonstrated the A2AR agonist’s protective effect, reducing pericyte apoptosis induced by OGD at the 18-hour time point. Scale bars: 50 μm. * Indicates *P* < 0.05, ** Indicates *P* < 0.01.

To explore the role of A2AR in pericyte apoptosis, cells were treated with the A2AR agonist CGS21680 or the A2AR antagonist SCH58261 under OGD conditions. As shown in Fig. 4A-D, TUNEL staining and flow cytometry revealed that, compared with that in the OGD group, the apoptotic index in the OGD + SCH group significantly increased. In contrast, the apoptotic index in the OGD + CGS group decreased (*P* < 0.05). We also determined the expression of the apoptosis-associated proteins BAX, Caspase 3 and BCL-2 via western blotting. As shown in Fig. 4F, G and I, western blot analysis further revealed that the protein expression levels of BAX and Caspase 3 were significantly lower in the OGD + CGS group than in the OGD group (*P* < 0.01). The protein expression levels of BAX and Caspase 3 were markedly greater in the OGD + SCH group than in the OGD group (*P* < 0.01). Compared with that in the OGD-control group, BCL-2 expression in the OGD + CGS group was significantly upregulated, whereas that in the OGD + SCH group was markedly downregulated (Fig. 4H, *P* < 0.05).

We also measured the protein expression of VEGFA and its receptor VEGFR2 in pericytes after CGS21680 or SCH58261 treatment under OGD conditions. Compared with that in the OGD group, VEGFA and VEGFR2 expression significantly increased in the OGD + CGS group (Fig. 4J and K, *P* < 0.01). Compared with that in the OGD group, VEGFA and VEGFR2 expression was significantly lower in the OGD + SCH group (Fig. 4J and K, *P* < 0.01). Moreover, the ELISA results confirmed that VEGFA expression was significantly greater in the OGD + CGS group than in the OGD group (Fig. 4E, *P* < 0.01).

### VEGFA suppression reverses the protective effect of A2AR against pericyte apoptosis under OGD conditions

**Fig. 5:**
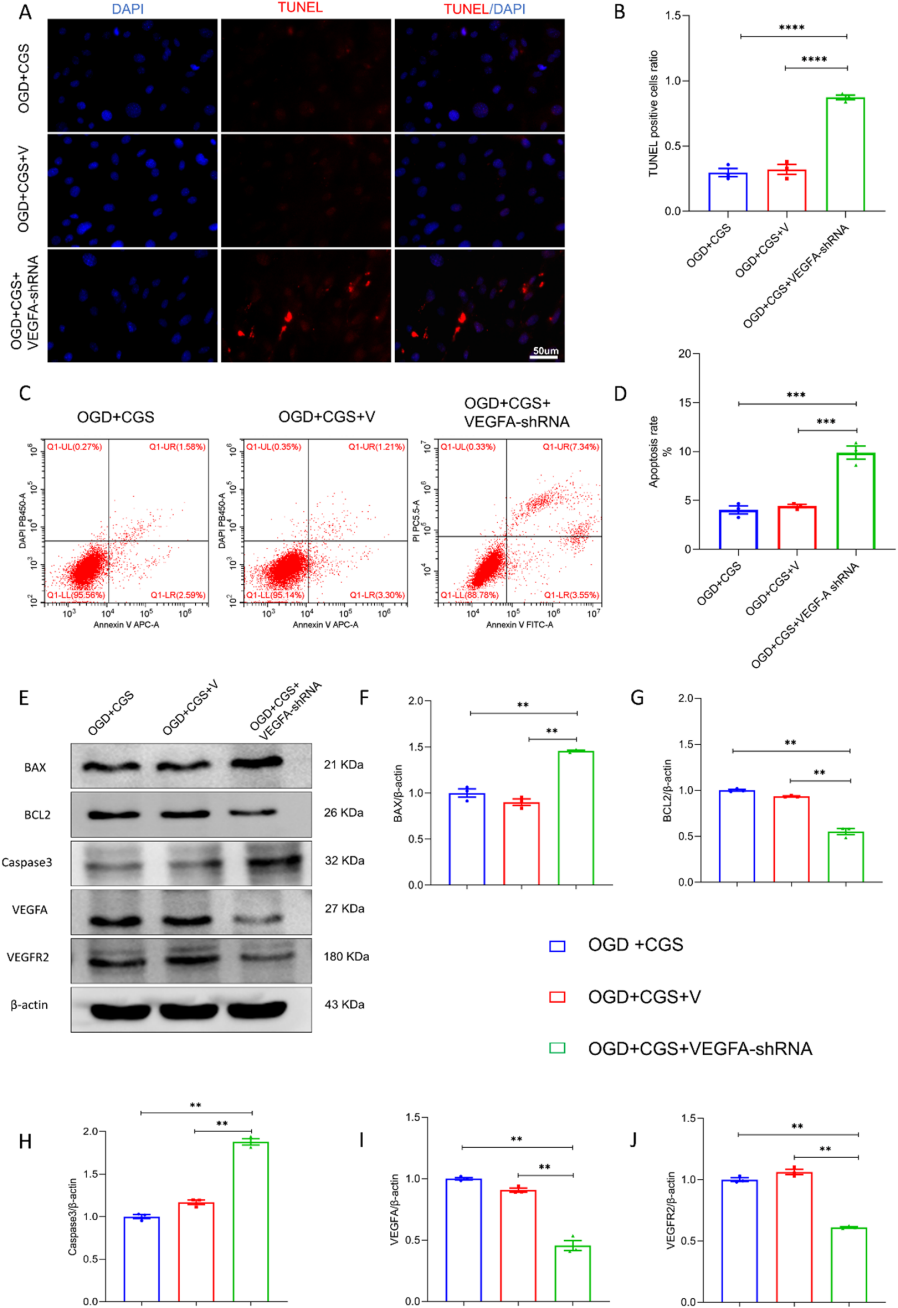
VEGFA involvement in A2AR-mediated pericyte apoptosis in response to oxygen-glucose deprivation. (**A**) This panel presents merged immunofluorescence images depicting TUNEL-positive apoptotic cells (red) and DAPI-stained nuclei (blue) in pericytes at 18 h of OGD. (**B**) Quantitative analysis of TUNEL-positive cell ratio at 18 h post-OGD reveals the relative extent of apoptosis among different treatment groups. (**C**-**D**) Flow cytometry measurements of pericyte apoptosis rate at 18 h under OGD conditions. (**E**-**J**) Western blot analysis depicts the protein expression levels of key apoptotic markers (BAX, BCL-2, Caspase3) and angiogenic factors (VEGFA and VEGFR2) in pericytes at 18 h post-OGD. Each experiment was performed with *n* = 3 replicates per group to ensure statistical reliability. Scale bars: 50 μm. * Indicates *P* < 0.05, ** Indicates *P* < 0.01.

To determine the contribution of VEGFA to pericyte apoptosis under OGD conditions, we utilized a shRNA-expressing lentiviral vector to downregulate VEGFA expression. The apoptotic response was then evaluated via TUNEL staining and flow cytometric analysis. Figure 5A-D shows a significant increase in the number of TUNEL-positive cells and the apoptosis rate in the OGD + CGS + VEGFA-shRNA group compared with the OGD + CGS + V control group (*P* < 0.01), indicating that VEGFA suppression augments pericyte apoptosis even in the presence of the A2AR agonist CGS21680.

Further analysis via western blotting was conducted to measure the levels of the proapoptotic proteins BAX and Caspase3, the antiapoptotic protein BCL-2, and components of the VEGFA-VEGFR2 signaling pathway in pericytes subjected to OGD (Fig. 5E-J). We found that the protein levels of BAX and Caspase3 were significantly elevated (*P* < 0.01) while the levels of BCL-2 and VEGFR2 were significantly reduced (*P* < 0.01) in the OGD + CGS + VEGFA-shRNA group compared with the OGD + CGS + V group. These findings suggest that the neuroprotective effect of A2AR activation is attenuated by VEGFA suppression. The reversal of the antiapoptotic effect of A2AR agonism upon VEGFA knockdown underscores the pivotal role of VEGFA in modulating pericyte survival under OGD conditions. Specifically, the shift in the balance between pro- and antiapoptotic protein expression in response to VEGFA suppression in the presence of CGS21680 highlights the interplay between VEGFA and A2AR in the regulation of pericyte apoptosis.

### A2AR protects against pericyte apoptosis via Rap1-ERK signaling under OGD conditions

**Fig. 6:**
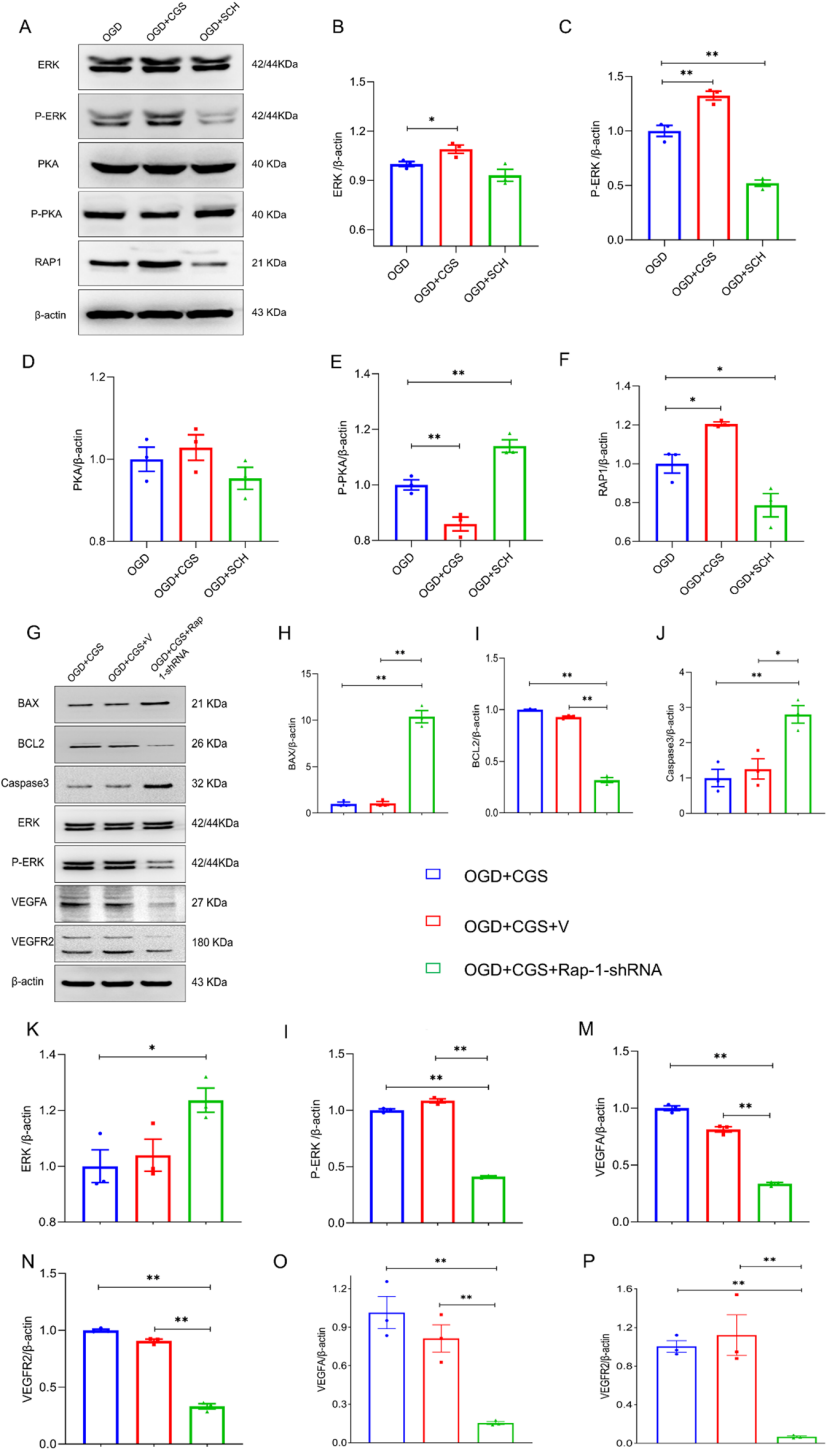
Rap1-ERK signaling participates in A2AR-mediated pericyte apoptosis in response to oxygen-glucose deprivation. (**A**-**F**) Western blot analysis was utilized to evaluate the protein levels of nuclear transcription factors (PKA, ERK, p-PKA and p-ERK) alongside angiogenic factors (VEGFA and VEGFR2) across the OGD group, OGD + CGS group, and OGD + SCH group at the 18-hour mark post-OGD induction. (**G**-**N**) Western blot assessment targeted apoptotic proteins (BAX, Caspase3, and BCL-2), nuclear transcription factors (ERK and p-ERK), and angiogenic factors (VEGFA and VEGFR2) in the OGD group, OGD + CGS + V group, and OGD + CGS + Rap-1-shRNA group under the same temporal conditions. (**O**-**P**) Q-RTPCR analysis was employed to quantify mRNA levels of VEGFA and VEGFR2 in the OGD group, OGD + CGS + V group, and OGD + CGS + Rap-1-shRNA group at 18 h post-OGD, providing insights into the transcriptional response to A2AR modulation and Rap-1 suppression. All experimental groups were treated under different conditions, and each was executed with triplicate replicates (*n* = 3) to ensure the reliability of the findings. * Indicates *P* < 0.05, ** Indicates *P* < 0.01.

Western blot analysis revealed significant upregulation of Rap-1, ERK, and phosphorylated ERK in pericytes treated with the A2AR agonist CGS21680 under OGD conditions compared with those in the OGD group (*P* < 0.05), as depicted in Fig. 6A-F. Conversely, the OGD + SCH group, which was treated with the A2AR antagonist SCH58261, presented significantly lower levels of Rap-1 and phosphorylated ERK than the OGD group (*P* < 0.05). PKA expression in the OGD + CGS group remained unchanged at 18 h after OGD, but the expression of phosphorylated PKA was reduced in the OGD + CGS group and increased in the OGD + SCH group compared to the OGD group (*P* < 0.05).

Further investigation using an shRNA to suppress Rap-1 expression revealed significant upregulation of the apoptotic proteins BAX and Caspase3 and downregulation of the antiapoptotic protein BCL-2 in the OGD + CGS + Rap-1-shRNA group compared with the OGD + CGS + V group (*P* < 0.01), as shown in Fig. 6G-N. Additionally, a marked reduction in the protein expression of phosphorylated ERK, VEGFA, and VEGFR2 was observed in the OGD + CGS + Rap-1-shRNA group compared with the OGD + CGS + V group (*P* < 0.01). Consistent with the western blot results, q-RT‒PCR analysis confirmed that VEGFA and VEGFR2 mRNA levels were also significantly decreased in the OGD + CGS + Rap-1-shRNA group at 18 h after OGD (Fig. 6O and P, *P* < 0.01). These data collectively suggest that A2AR agonists promote the expression of VEGFA and its receptor VEGFR2, thereby reducing pericyte apoptosis under OGD conditions by activating the Rap1-ERK signaling pathway.

## Discussion

In this study, we confirmed the occurrence of brain pericyte apoptosis in the context of CCH. Elevated VEGFA expression is likely linked to CCH-induced pericyte apoptosis, as evidenced by both in vivo and in vitro studies. A2AR agonists may provide neuroprotection by inhibiting pericyte apoptosis in CCH, whereas antagonists may aggravate it. Increased VEGFA expression contributes to the neuroprotective effect of A2AR by mitigating pericyte apoptosis. Notably, A2AR significantly increases VEGFA and VEGFR2 expression via the Rap1-ERK signaling pathway, thus inhibiting apoptosis under low-glucose and hypoxic conditions.

### Elevated VEGFA expression is potentially associated with pericyte apoptosis triggered by CCH

CCH results in an inadequate blood supply to the brain, leading to metabolic disorders and a cascade of pathophysiological changes, including neuronal loss and aberrant apoptosis^30^. Research has revealed a reduction in pericyte number due to apoptosis in CCH, resulting in diminished BBB integrity. Pericytes help maintain the integrity of the blood-brain barrier and reduce the paracellular permeability of endothelial cells by promoting the formation of tight junctions and maintaining low levels of endocytosis in the brain endothelium^31^. Pericytes also respond to cytokines, triggering the release of pro-inflammatory molecules, leading to the breakdown of the blood-brain barrier in vitro, and inducing the activation of pro-inflammatory states in astrocytes, endothelial cells, and microglia (innate immune cells of the brain). Importantly, pericytes are crucial in cerebral vascular dysfunction in patients with neurodegenerative diseases^32^. In addition, the absence of pericytes can have many effects on vascular function, such as impaired angiogenesis, increased vascular permeability, decreased vascular regeneration ability, and dysfunction of vascular contraction and relaxation^33^. Bilateral carotid artery ligation method for pericytes of rat brain cortex decreased significantly after 4 weeks of modeling, while the surrounding structures of microvascular lacking pericellular coverage are swollen and the lumen is twisted, causing a decrease in cerebral blood flow^34^. Consistent with previous results, we confirmed the occurrence of pericyte apoptosis in a rat CCH model through immunofluorescence. Our in vitro studies, which involved TUNEL staining, flow cytometry, and western blot analysis of the expression of apoptotic markers (BAX, Caspase3, and BCL-2), further confirmed the occurrence of pericyte apoptosis under OGD conditions. TUNEL staining revealed a significant increase in apoptotic cells at 12 and 18 h after OGD exposure. Flow cytometry revealed peak apoptosis at 6, 12 and 18 h. Western blotting revealed upregulation of the proapoptotic proteins BAX and Caspase 3 and downregulation of the antiapoptotic protein BCL-2, particularly at 12 and 18 h after OGD exposure. Collectively, both the in vivo and in vitro findings substantiate that pericyte apoptosis occurs in CCH.

In addition, we evaluated the protein levels of VEGFA and its receptor VEGFR2 in the corpus callosum of CCH model rats via western blotting and ELISA. VEGFA, crucial for vascular permeability and angiogenesis, has been implicated in neurodegeneration^35^, traumatic brain injury^36^, and ischemia^37^. Previous results have shown that VEGFA protein expression significantly increases from 24 h to 21 days after surgery in rats with CCH^38^. The VEGFA/VEGFR signaling pathway activation alleviates cognitive impairment induced by chronic hypoperfusion^39^. The inhibition of VEGFA and its receptor VEGFR2 has been proven to ameliorate cerebral ischemia-induced BBB dysfunction^40,41^. Our findings revealed an initial decrease followed by a significant increase in VEGFA and VEGFR2 protein expression after CCH induction, mirroring the changes in the expression of the antiapoptotic BCL-2 protein in cultured pericytes exposed to OGD. These results suggest a close association between VEGFA and pericyte apoptosis in CCH. Similarly, recent studies have shown that VEGFA mitigates neural damage in CCH by inhibiting apoptosis and autophagy^42^, potentially preventing cognitive decline^43^.

### A2AR-mediated VEGFA upregulation reduces pericyte apoptosis in chronic cerebral hypoperfusion

Our previous research focused on elucidating the mechanisms by which A2AR regulates neuroinflammatory responses associated with CCH, with the ultimate goal of identifying strategies to alleviate the ensuing white matter damage in the brain. Our previous findings indicated that the absence of A2AR exacerbates white matter damage induced by CCH^18^. Intriguingly, the silencing of A2AR in microglia has been shown to ameliorate this damage. In contrast, the opposite effect is observed when A2AR is silenced in bone marrow-derived cells, highlighting the predominant neuroprotective role of bone marrow-derived cells^19^. Given their presence in the blood circulation, bone marrow-derived cells are hypothesized to enter the CNS via the compromised BBB. The modulation of BBB permeability by purinergic receptors is notably complex and, at times, paradoxical^22^. Specifically, the activation of A2AR is generally neuroprotective, primarily because it mitigates BBB damage and increases its integrity^44,45^. However, studies have reported that A2AR activation can also exacerbate BBB damage, increasing its permeability^20,23^. The role of pericytes within the BBB has garnered increasing attention in recent years. Yet, the ability of modulating A2AR expression to regulate pericyte function and thus preserve BBB integrity remains understudied. In this study, we utilized TUNEL staining and flow cytometry to confirm that the A2AR agonist CGS21680 significantly suppresses pericyte apoptosis both in vivo and in vitro. Western blot analysis further revealed that CGS21680 notably reduced the proapoptotic proteins BAX and Caspase 3 while concurrently increasing the expression of the antiapoptotic protein BCL-2. Conversely, the A2AR antagonist SCH58261 had the opposite regulatory effect. Thus, A2AR protects against BBB damage induced by CCH by inhibiting pericyte apoptosis.

Additionally, our study showed that the A2AR agonist CGS21680 significantly upregulated the expression of VEGFA and its receptor VEGFR2 in pericytes under OGD conditions in vitro. Conversely, the A2AR antagonist SCH58261 had the opposite effect. Similar changes were observed in an in vivo model, with CGS21680 leading to a significant increase in VEGFA and VEGFR2 expression in the corpus callosum of rats with CCH, especially as the duration of cerebral ischemia increased. Interestingly, SCH58261 treatment resulted in a pronounced decrease in the expression of VEGFA and VEGFR2. In contrast, no significant difference in VEGFA or VEGFR2 expression was observed between the 2VO-saline group and the 2VO-CGS group at any time point after CCH, suggesting a complex interplay of regulatory mechanisms in vivo. The discrepancy between the in vitro and in vivo findings led us to hypothesize that multiple pathways may be involved in regulating VEGFA and VEGFR2 expression in CCH model rats.

To reinforce the connection between VEGFA and A2AR-mediated regulation of pericyte apoptosis under OGD conditions, we used an adenoviral vector to interfere with VEGFA gene expression, successfully suppressing VEGFA protein expression in transfected pericytes. Subsequent results demonstrated that the neuroprotective effects of A2AR activation, specifically its inhibition of pericyte apoptosis, were significantly counteracted by the knockdown of VEGFA. These findings collectively highlight the impact of A2AR-induced VEGFA upregulation on the inhibition of pericyte apoptosis, particularly in CCH. The modulation of the VEGFA pathway by A2AR activation represents a potential mechanism underlying the neuroprotective effect of A2AR in CCH.

### A2AR activation and VEGFA expression in apoptotic pericytes under OGD conditions: the role of the Rap1-ERK pathway

A2AR, a G protein-coupled receptor (GPCR), is known to activate adenylate cyclase, leading to increased cyclic adenosine monophosphate (cAMP) levels and subsequent activation of protein kinase A (PKA). PKA, in turn, modulates gene expression by phosphorylating cAMP response element-binding protein (CREB)^46^. However, our study revealed no significant increase in PKA or phosphorylated PKA levels in pericytes after OGD, which is inconsistent with previous findings. In addition to CREB, A2AR can activate the expression of numerous signaling molecules and transcription factors to modulate cellular activity. These include nuclear factor kappa B (NF-κB), hypoxia-inducible factor 1 (HIF-1), mitogen-activated protein kinase/extracellular signal-regulated kinase (MAPK/ERK), Ras-related protein 1 (Rap-1), signal transducer and activator of transcription 3 (STAT3), phosphoinositide 3-kinase (PI3K), protein kinase B (AKT), and mammalian target of rapamycin (mTOR), among others. Notably, Rap-1, a small G protein and member of the Ras family^47^, interacts with ERK^48^, reciprocally activating and regulating cellular pathophysiology. Our results corroborate the findings of previous work showing that A2AR upregulates the expression of VEGFA^49,50^. However, the mechanism underlying this upregulation of VEGFA expression remains elusive. We discovered a significant increase in Rap-1 expression following A2AR activation in OGD-exposed pericytes. Using a shRNA to suppress Rap-1, we found that Rap-1 inhibition reversed the antiapoptotic effects of A2AR activation. Additionally, ERK phosphorylation and the protein expression of VEGFA and VEGFR2 were markedly reduced in OGD-exposed pericytes after Rap-1 suppression, indicating that the Rap1-ERK pathway plays a role in A2AR-mediated VEGFA upregulation to inhibit pericyte apoptosis under OGD conditions. While there is little evidence for a connection between A2AR activation and Rap-1 expression, our study confirms that this connection exists, providing a novel avenue for understanding the role of A2AR in pericyte survival in the context of cerebral ischemia.

## Conclusion

In conclusion, our research demonstrates that the activation of adenosine A2AR attenuates pericyte apoptosis in CCH, potentially by increasing the activity of the VEGFA signaling pathway. Importantly, our findings suggest that the Rap1-ERK pathway may be instrumental in mediating the A2AR-induced upregulation of VEGFA expression in pericytes following CCH. These insights underscore the potential of A2AR agonists in conferring neuroprotection in CCH and the need for further research on their therapeutic efficacy.

## Electronic supplementary material

Below is the link to the electronic supplementary material.

Supplementary Material 1

## Data Availability

The datasets used and/or analysed during the current study available from the corresponding author on reasonable request.
