# Supplementary material for: Upregulation of VEGFA through the adenosine A2A receptor is a crucial pathway for inhibiting pericyte apoptosis in chronic cerebral hypoperfusion
Source: Sci Rep. 2025 Jul 4;15:23955. doi: 10.1038/s41598-025-08407-2 (PMC12227720; doi:10.1038/s41598-025-08407-2)

**Supplementary materials**

Figure S 2A:

VEGFA
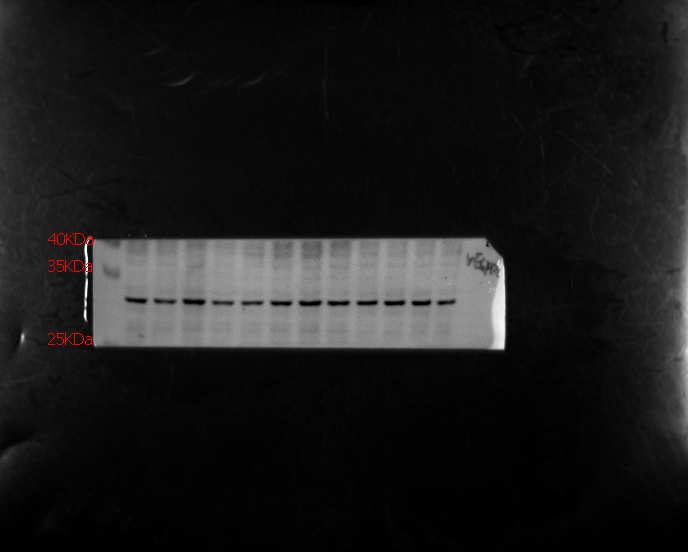


VEGFR2


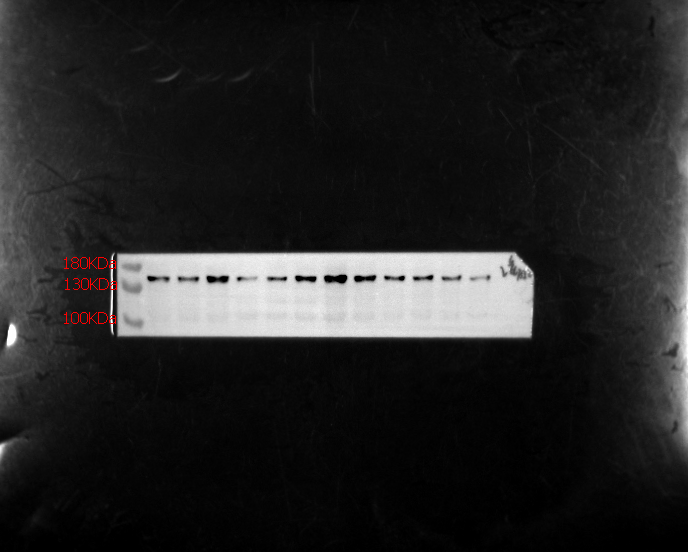


β-Actin


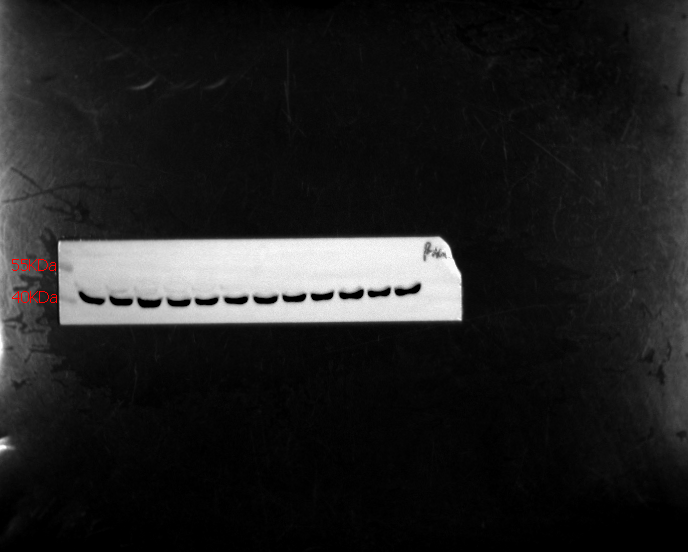


Figure S 3E:

BAX


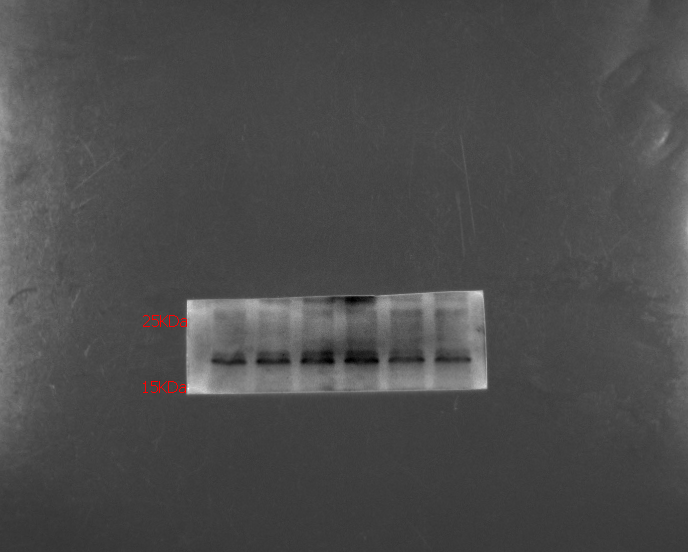


BCL2


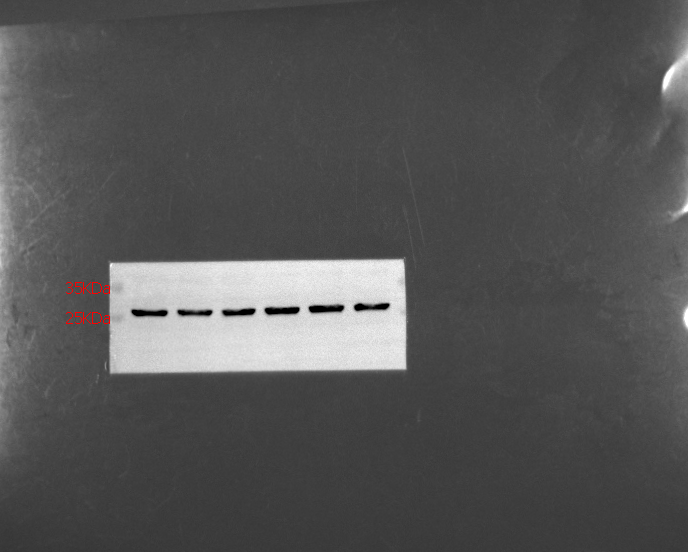


Caspase3


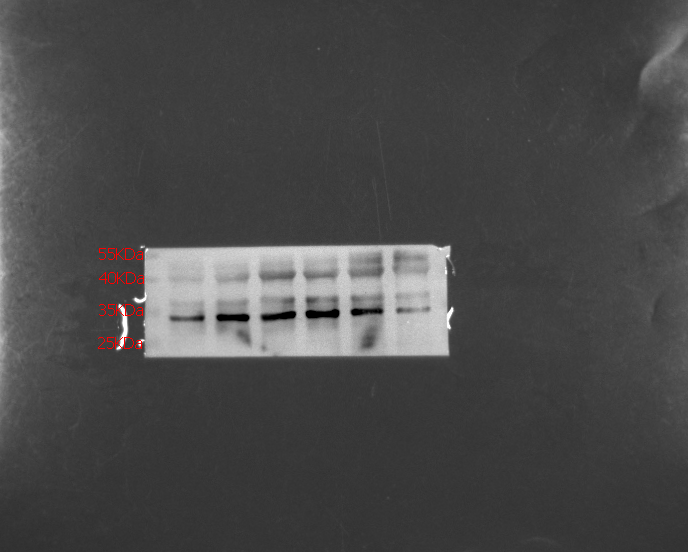


Tubulin


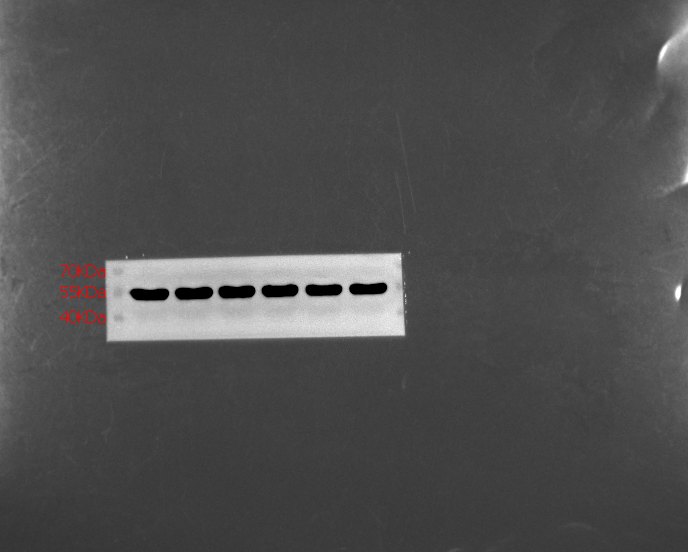


VEGFA


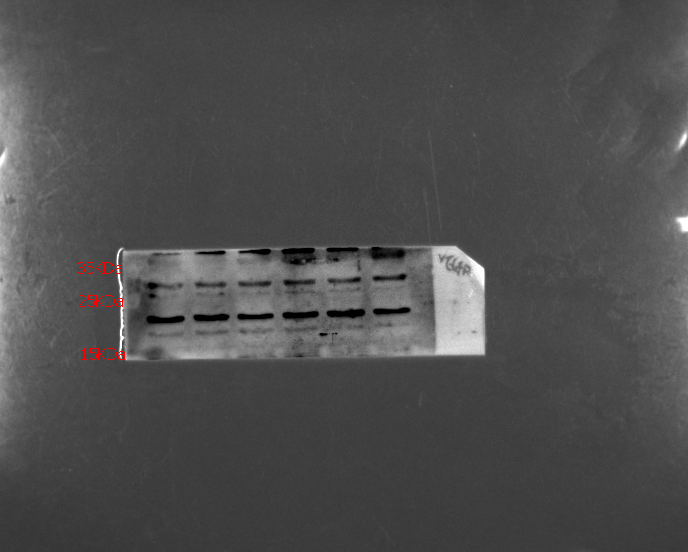


β-Actin


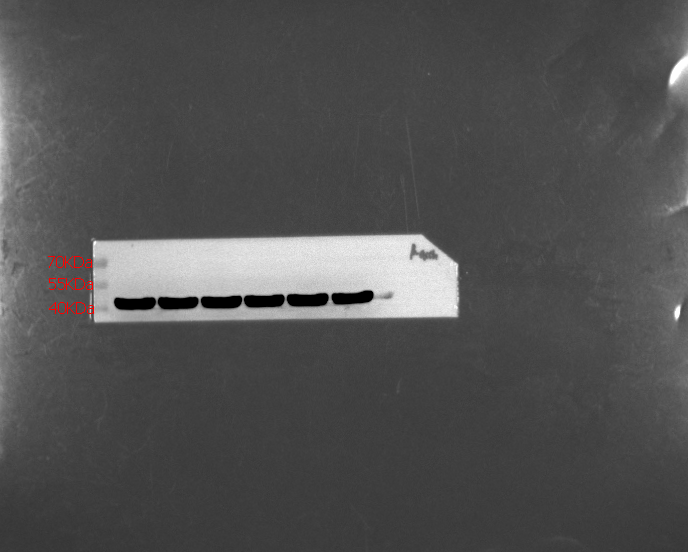


Figure S 4F:

BAX


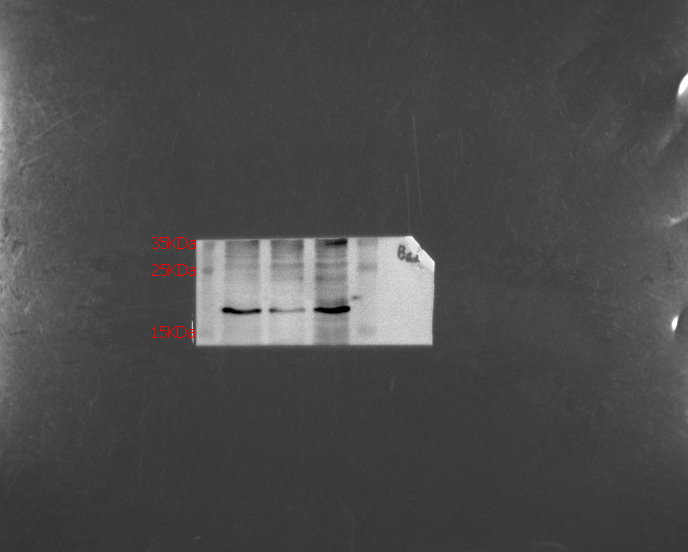


BCL2


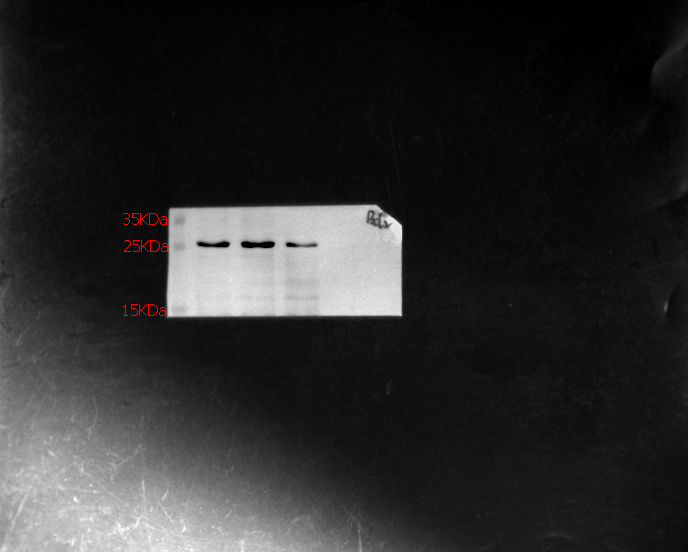


Caspase3


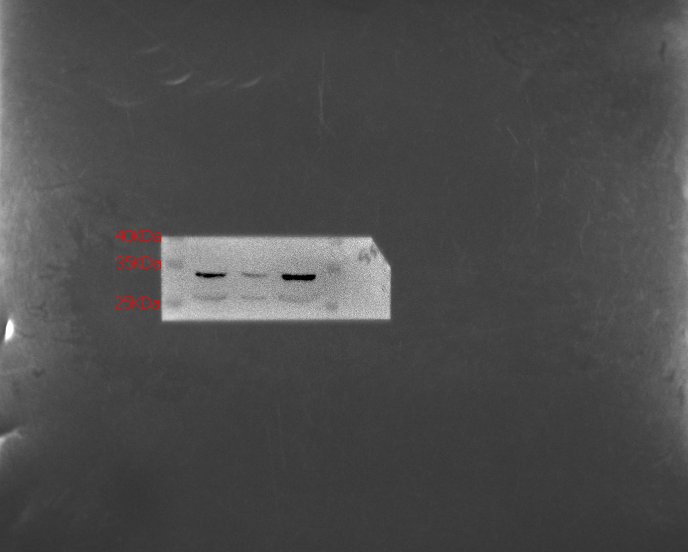


VEGFA


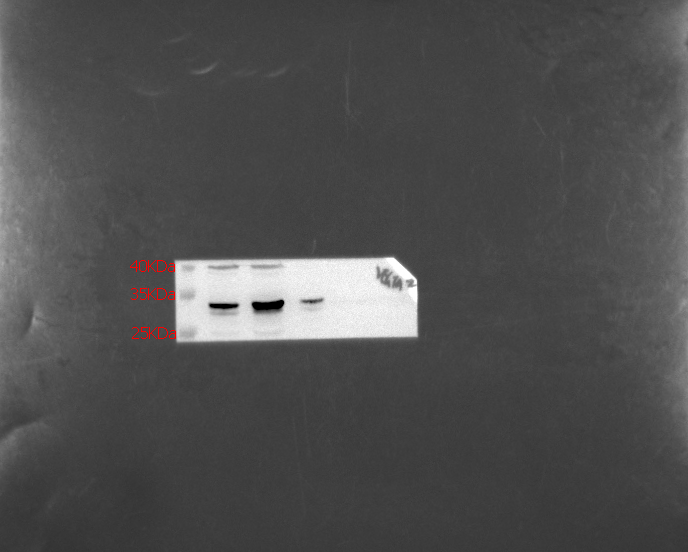


VEGFR2


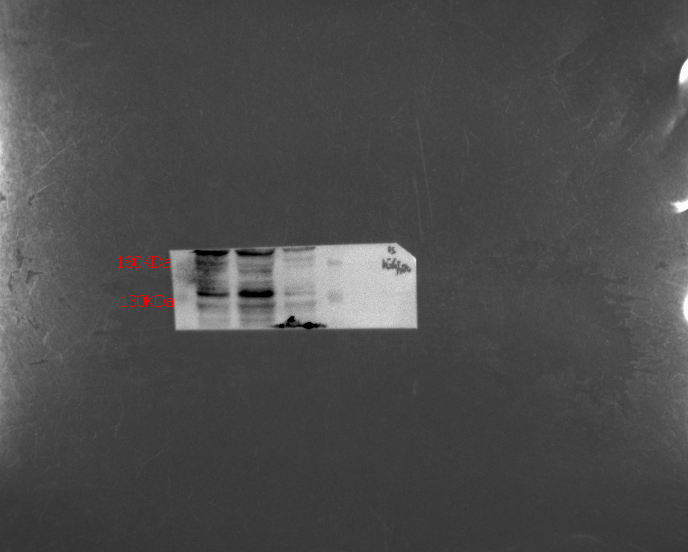


β-Actin


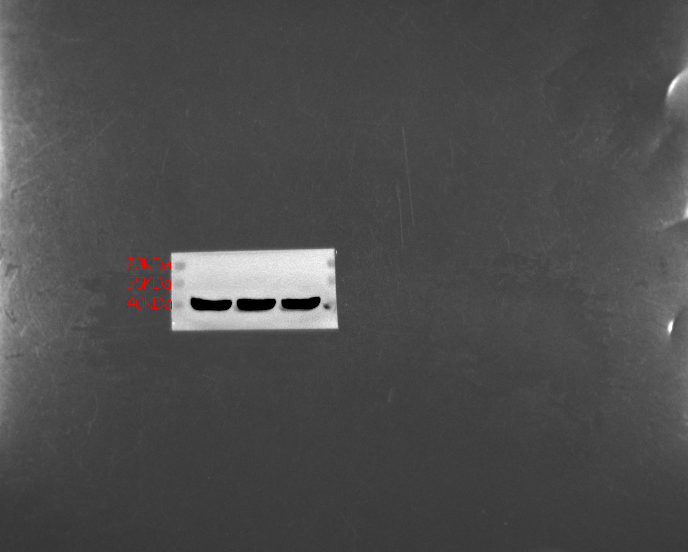


Figure S 5E:

BAX


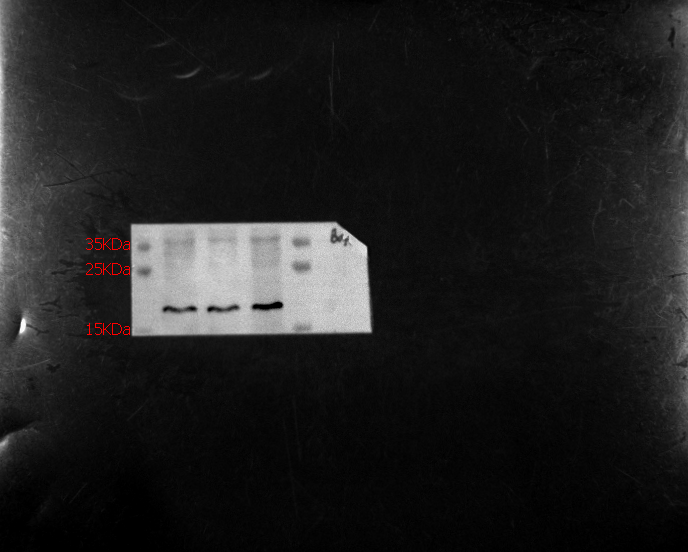


BCL2


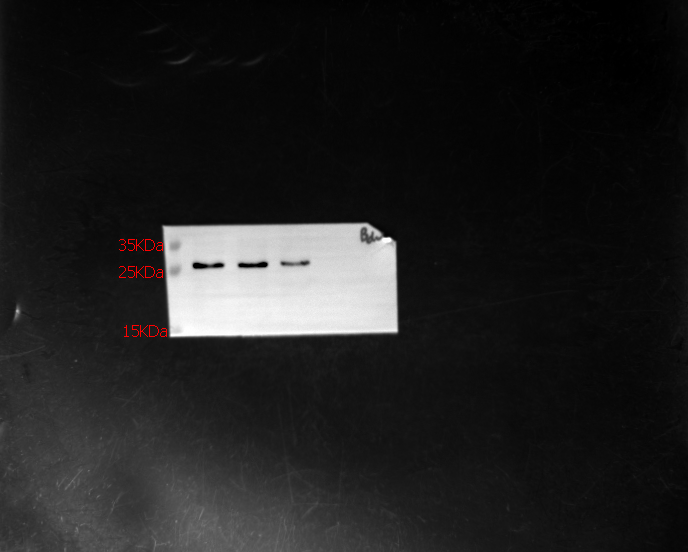


Caspase3


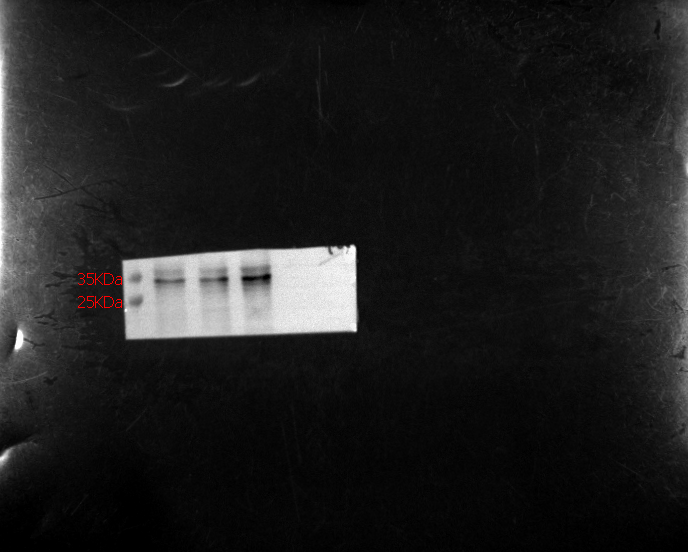


VEGFA


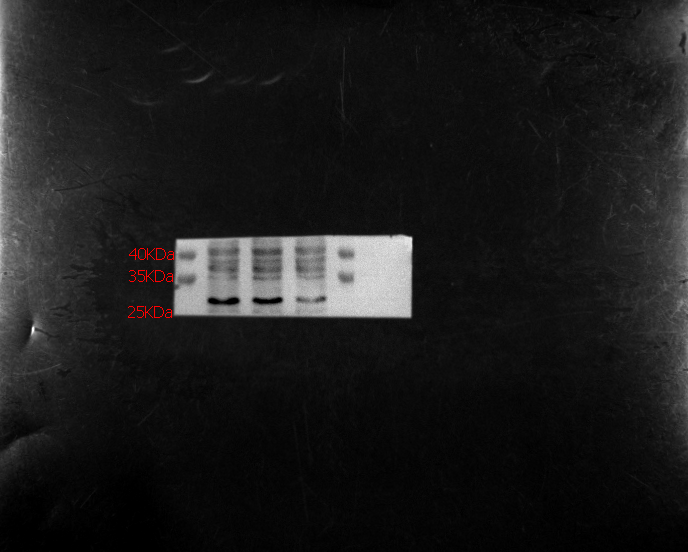


VEGFR2


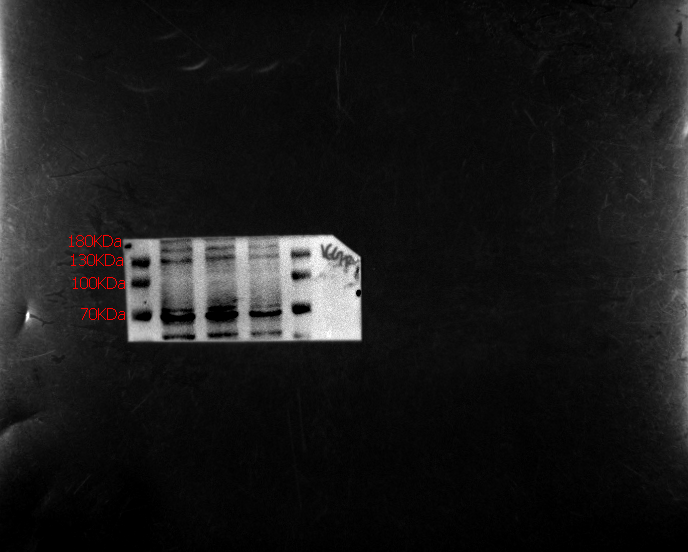


β-Actin


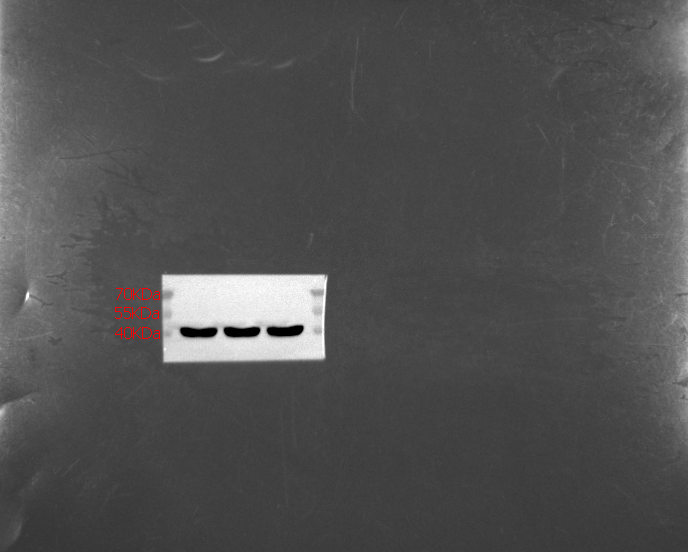


Figure S 6A:

ERK


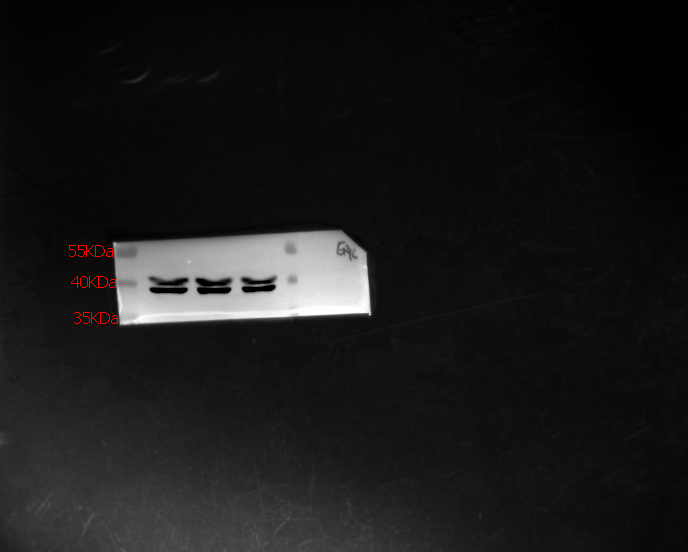


P-ERK


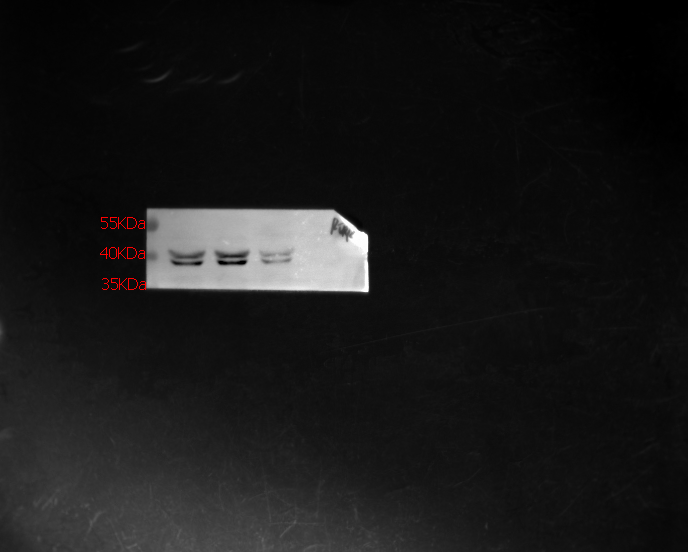


PKA


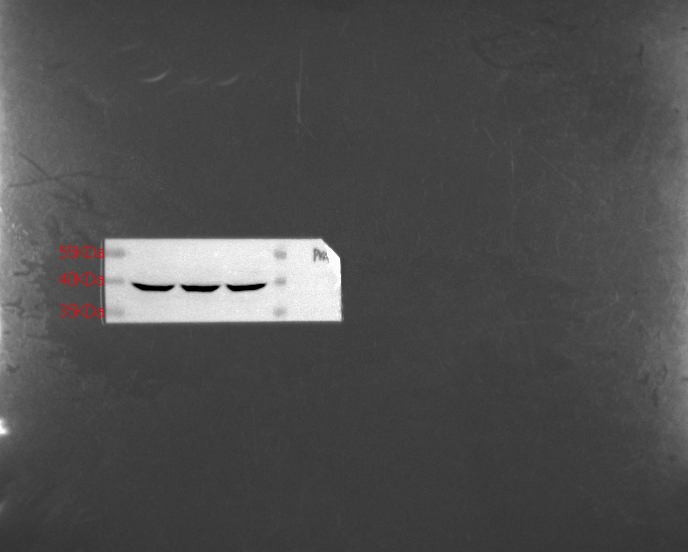


P-PKA


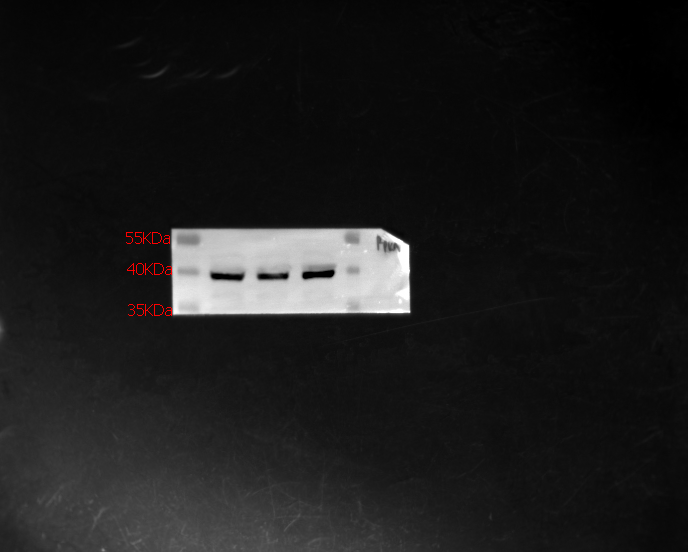


RAP-1


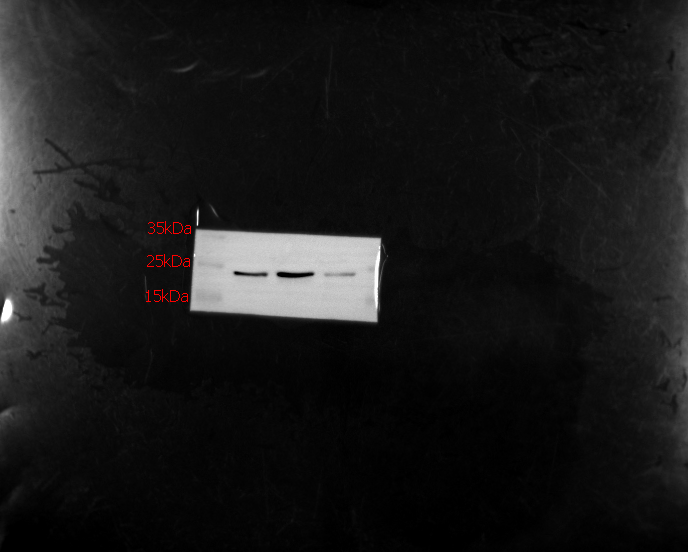


β-Actin


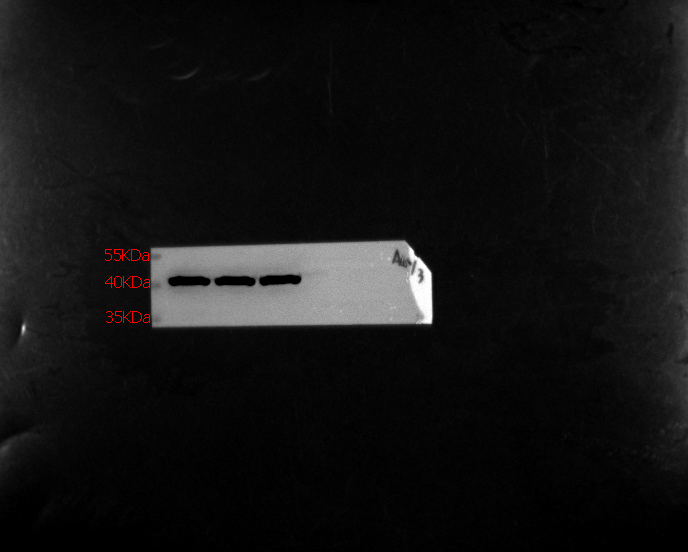


Figure S 6G:

BAX


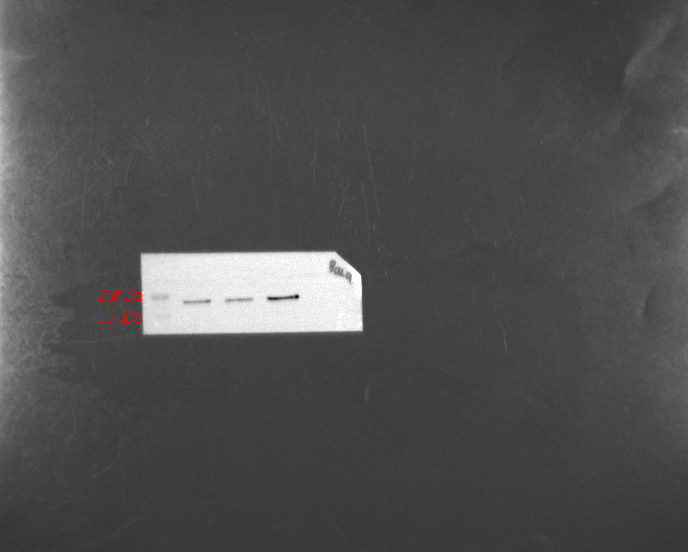


BCL2


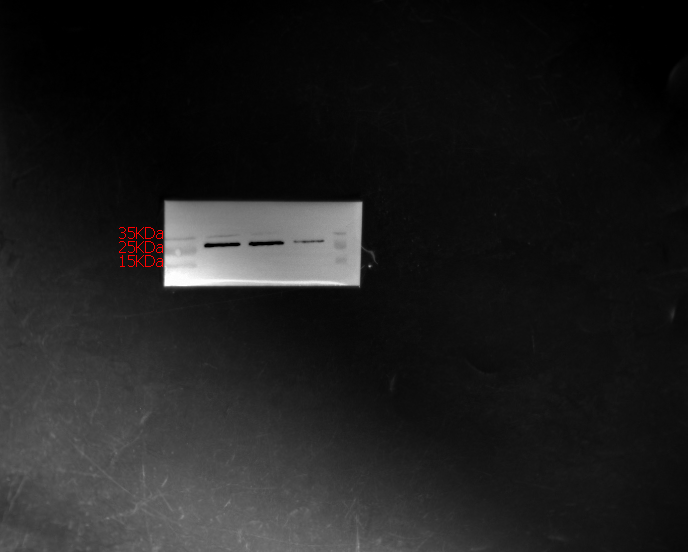


Caspase3


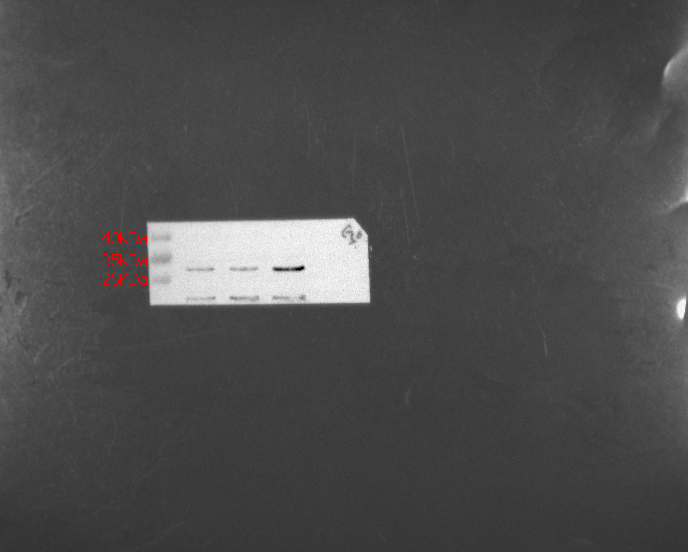


ERK


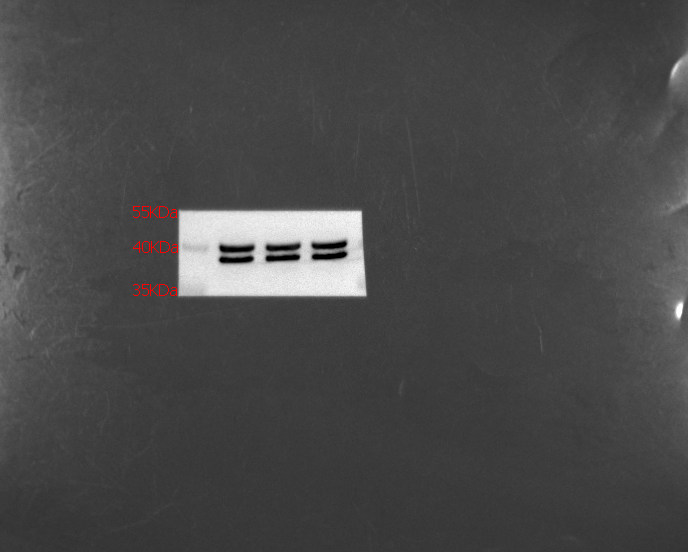
P-ERK


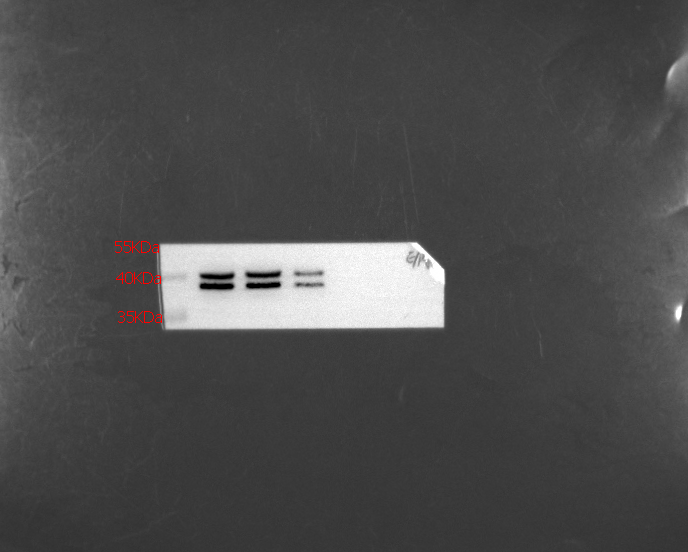


VEGFA


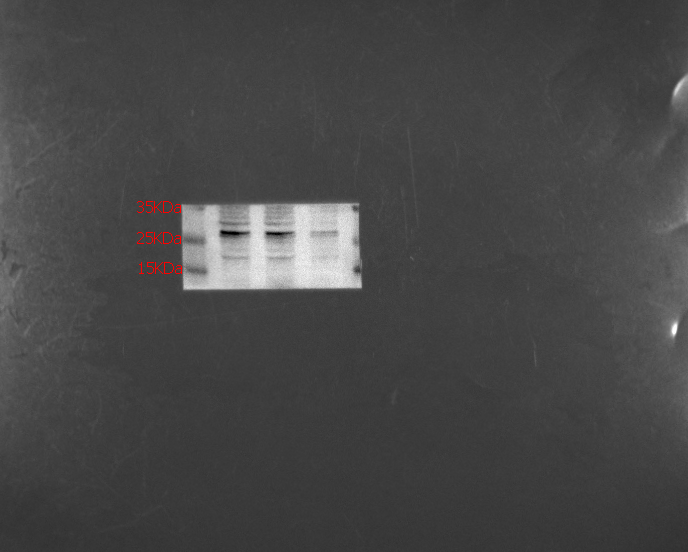


VEGFR2


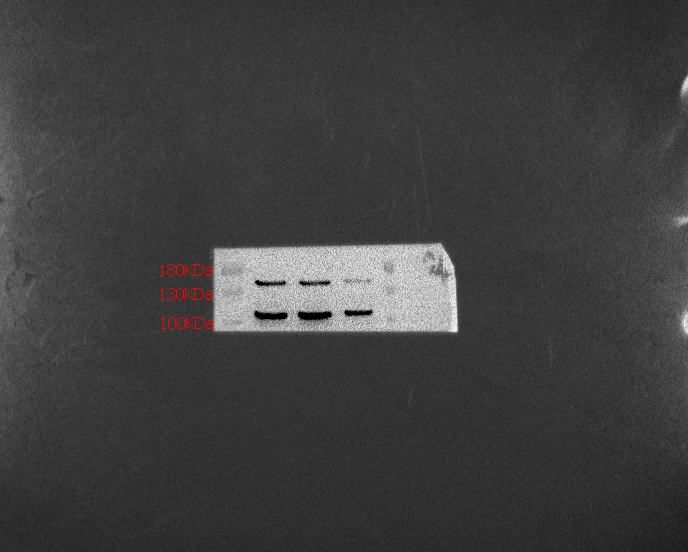


β-Actin


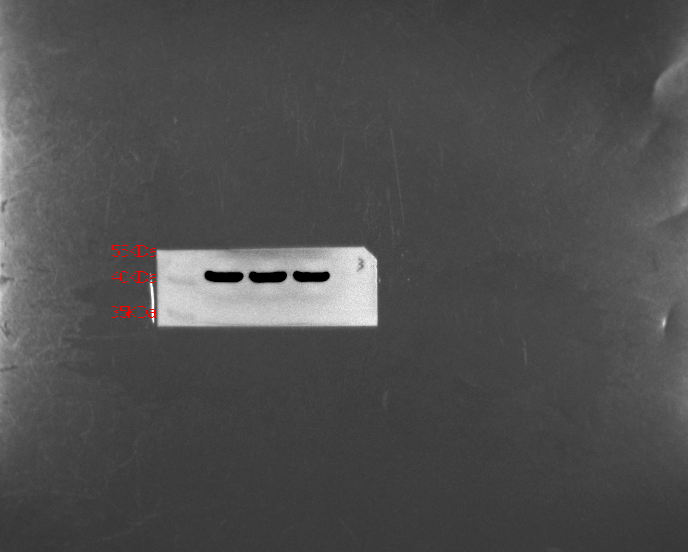

Supplement: Supplementary file 1 — Supplementary Material 1 [file 41598_2025_8407_MOESM1_ESM.docx]
